# ADH1B, the adipocyte-enriched alcohol dehydrogenase, plays an essential, cell-autonomous role in human adipogenesis

**DOI:** 10.1073/pnas.2319301121

**Published:** 2024-06-05

**Authors:** Jérémie Gautheron, Solaf Elsayed, Valeria Pistorio, Sam Lockhart, Jamila Zammouri, Martine Auclair, Albert Koulman, Sarah R. Meadows, Marie Lhomme, Maharajah Ponnaiah, Redouane Si-Bouazza, Sylvie Fabrega, Abdelaziz Belkadi, Qatar Genome Project, Jean-Louis Delaunay, Tounsia Aït-Slimane, Bruno Fève, Corinne Vigouroux, Tawhida Y. Abdel Ghaffar, Stephen O’Rahilly, Isabelle Jéru

**Affiliations:** ^a^Centre de Recherche Saint-Antoine, Sorbonne Université-Inserm, Paris 75012, France; ^b^Foundation for Innovation in Cardiometabolism and Nutrition, Paris 75013, France; ^c^Medical Genetics Department, Faculty of Medicine, Ain Shams University, Cairo 11566, Egypt; ^d^Wellcome Trust-Medical Research Council Institute of Metabolic Science, University of Cambridge, Cambridge CB2 1TN, United Kingdom; ^e^Omics Lipidomics, Foundation for Innovation in Cardiometabolism and Nutrition, Paris 75013, France; ^f^Data sciences unit, Foundation for Innovation in Cardiometabolism and Nutrition, Paris 75013, France; ^g^Viral Vector and Gene Transfer Platform, Structure Federative de Recherche Necker, Université Paris Cité, Paris 75015, France; ^h^Bioinformatics Core, Weill Cornell Medicine-Qatar, Education City, Doha 24144, Qatar; ^i^Qatar Genome Program, Foundation Research, Development and Innovation, Qatar Foundation, Doha 24144, Qatar; ^j^Centre National de Référence des Pathologies Rares de l’Insulino-Sécrétion et de l’Insulino-Sensibilité, Service de Diabétologie et Endocrinologie de la Reproduction, Hôpital Saint-Antoine, Assistance Publique-Hôpitaux de Paris, Paris 75012, France; ^k^Yassin Abdelghaffar Center for Liver Disease and Research, Cairo 11566, Egypt; ^l^Medical Genetics Unit, Biology, Genomics and Hygiene Medical-University Department, Pitié-Salpêtrière Hospital, Sorbonne Université, Assistance Publique-Hôpitaux de Paris, Paris 75013, France

**Keywords:** alcohol dehydrogenase 1B, adipocyte differentiation, 9-cis retinoic acid, human adipose stem cells, ADH1B

## Abstract

Alcohol dehydrogenase 1B (ADH1B) is a primate-specific enzyme which, uniquely among the ADH class 1 family, is highly expressed both in adipose tissue and liver. Its expression in adipose tissue is reduced in obesity and increased by insulin stimulation. Interference with *ADH1B* expression has also been reported to impair adipocyte function. To better understand the role of ADH1B in adipocytes, we used CRISPR/Cas9 to delete *ADH1B* in human adipose stem cells (ASC). Cells lacking ADH1B failed to differentiate into mature adipocytes manifested by minimal triglyceride accumulation and a marked reduction in expression of established adipocyte markers. As ADH1B is capable of converting retinol to retinoic acid (RA), we conducted rescue experiments. Incubation of ADH1B-deficient preadipocytes with 9-cis-RA, but not with all-transretinol, significantly rescued their ability to accumulate lipids and express markers of adipocyte differentiation. A homozygous missense variant in *ADH1B* (p.Arg313Cys) was found in a patient with congenital lipodystrophy of unknown cause. This variant significantly impaired the protein’s dimerization, enzymatic activity, and its ability to rescue differentiation in ADH1B-deficient ASC. The allele frequency of this variant in the Middle Eastern population suggests that it is unlikely to be a fully penetrant cause of severe lipodystrophy. In conclusion, ADH1B appears to play an unexpected, crucial and cell-autonomous role in human adipocyte differentiation by serving as a necessary source of endogenous retinoic acid.

## Significance

The class I alcohol dehydrogenase (ADH) family, while best known for liver ethanol metabolism, metabolizes various other alcohols. Among them, ADH1B is exclusively found in primates and is unique among this family in also being highly expressed in fat tissue, where its role is unclear. Human adipose stem cells lacking ADH1B failed to differentiate into adipocytes. This was partially rescued either by reexpression of ADH1B or by cell incubation with 9-cis retinoic acid (9-cis RA), but not its alcohol precursor, all-transretinol. 9-cis RA activates the retinoid X receptor, the heterodimeric partner of PPARγ, the master regulator of adipogenesis. In conclusion, ADH1B in human adipocytes appears to be a necessary source of 9-cis RA required to support adipogenesis.

The members of the alcohol dehydrogenase (ADH) enzymatic family metabolize a wide variety of substrates, including alcohols, hydroxysteroids, and lipid peroxidation products (1, 2). In humans, the class I ADHs, which include three closely homologous proteins active as homo- or heterodimers, are encoded by the *ADH1A*, *ADH1B*, and *ADH1C* genes (1, 2). *ADH1A* and *ADH1C* are exclusively expressed in the liver (1). In contrast, *ADH1B*, which is found only in primates, is highly expressed in both adipose tissue and liver (1). The three isoforms can convert a range of alcohol products to their respective aldehydes. Overall, the amino acid sequence identity between the three isoforms is ~93%. However, in the substrate binding pocket, it falls to ~60% (2), which explains why the substrate preferences of the three isoenzymes have some distinct characteristics (3, 4). Their most well-known function is their role in the conversion of ethanol to acetaldehyde in the liver, with variation affecting this gene cluster being strongly associated with alcohol tolerance and alcohol consumption in humans (5–7).

The abundant expression of *ADH1B* in human adipose tissue has drawn the attention of some investigators. Winnier et al. (8) and Kerr et al. (9) both reported a strong inverse association between *ADH1B* mRNA levels in adipose tissue biopsies with measures of human adiposity. Morales et al. confirmed these findings in multiple ethnicities, reported that ADH1B protein levels in human isolated adipocytes were increased by insulin and showed that partial knockdown of *ADH1B* in human preadipocytes resulted in an impairment of their ability to differentiate (10), a finding also reported by Kerr et al. (9).

To more definitively evaluate the role of this enzyme in human adipocyte biology, we used CRISPR-Cas9 to delete the gene in two independent lines of human adipose stem cells (ASC) and measured the impact of this on differentiation. Having demonstrated the severe impact of ADH1B deficiency on adipogenesis, we undertook rescue experiments to attempt to identify a product of ADH1B enzymatic activity that might play a critical, cell-autonomous role in human adipogenesis.

## Results

### Marked Impairment of Adipocyte Differentiation by Knock Out of *ADH1B* in Human ASC.

**Fig. 1.**
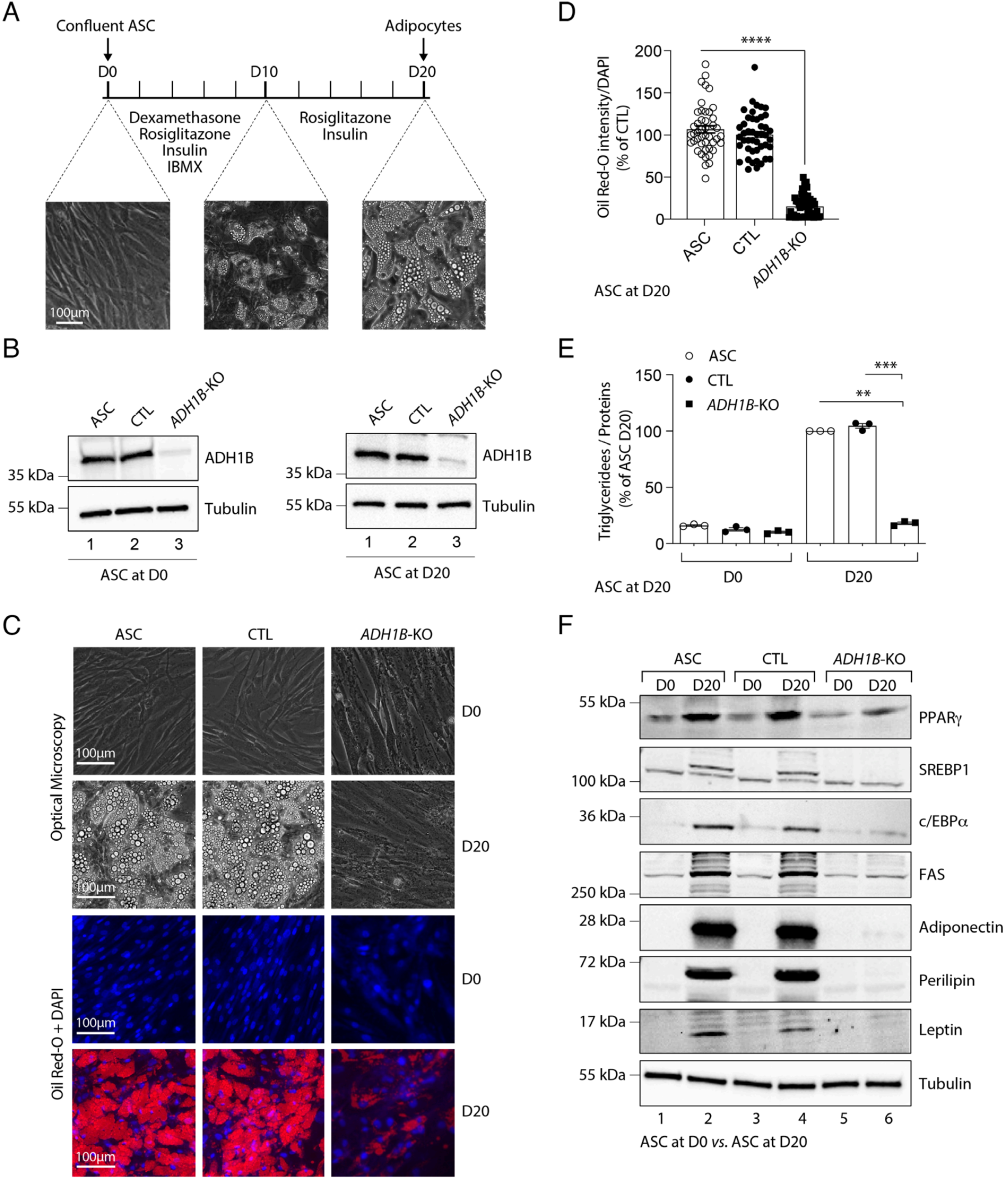
*ADH1B* deficiency in ASC alters lipid droplet formation and triglyceride content. Data were obtained in ASC, ASC with a CRISPR-Cas9-mediated *ADH1B*-knockout (KO), and ASC transduced with a Cas9/scramble gRNA plasmid corresponding to control (CTL) cells. (*A*) Timeline representation of the ASC differentiation process using a hormonal cocktail. IBMX: 3-isobutyl-1-methylxanthine; D0: day 0 (undifferentiated state); D10: day 10; D20: day 20. (*B*) ADH1B expression in ASC during adipocyte differentiation and validation of *ADH1B* KO in ASC at D0 and at D20. Numbers on the left correspond to molecular weight markers (kDa). Western blot images are representative of three independent experiments. (*C*) Adipocyte differentiation assessed by Oil Red-O lipid staining. ASC preadipocytes were studied during adipocyte differentiation for 20 d. First and second lines: representative pictures of cell dishes by optical microscopy. Images are representative of three independent experiments. Third and fourth lines: representative images of fluorescence microscopy after staining of intracellular lipids (Oil Red-O, red) and nuclei (DAPI, blue). Images are representative of three independent experiments. (*D*) Quantification of Oil Red-O fluorescence normalized to DNA content (DAPI). Results are expressed as means ± SEM of three independent experiments. (*E*) Intracellular triglyceride contents were measured at D0 and D20 in ASC, CTL, and *ADH1B* KO cells. The measurements are representative of three independent experiments. (*F*) Protein expression of adipocyte markers obtained by western blotting during in vitro adipocyte differentiation of ASC cells at D0 and D20. Numbers on the left correspond to molecular weight markers (kDa). Western blot images are representative of three independent experiments. PPARγ: peroxisome proliferator-activated receptor-gamma; C/EBPα: CCAAT/enhancer-binding protein-alpha; SREBP-1c: sterol regulatory element-binding protein-1c; FAS: fatty acid synthase. Numbers on the left correspond to molecular weight markers (kDa). Western blot images are representative of three independent experiments. **P < 0.01, ***P < 0.001, ****P < 0.0001, n.s. designates nonspecific bands.

Inspection of data relating to human adipose tissue on the GTEX portal (https://gtexportal.org/home/) reveals *ADH1B* to be one of the 50 most highly expressed genes. To better understand its role in this tissue, we proceeded to delete the *ADH1B* gene from human ASC (Fig. 1 *A* and *B*). A custom-designed guide RNA (gRNA)/Cas9 expression vector targeting the fourth exon of *ADH1B* was inserted into lentiviral particles before ASC infection. A Cas9/scramble gRNA plasmid was used as a control (CTL). Sanger sequencing of *ADH1B* exon 4 in genomic DNA from KO ASC revealed 98% of on-target indels, which were not found in CTL cells. (*SI Appendix*, Figs. S1 and S2). The efficiency of *ADH1B* KO was confirmed by western blot analysis, which showed a >90 % reduction of ADH1B expression at D0 and after 20 d of differentiation (D20) (Fig. 1*B*).

When compared to CTL cells, *ADH1B* KO cells had markedly less lipid droplet formation (Fig. 1 *C* and *D*) and triglyceride content (Fig. 1*E*) (both *P* < 0.0001) after 20 d of differentiation. At D20, the levels of PPARγ, C/EBPα, and SREBP1c proteins, key transcription factors associated with adipogenesis, were all markedly reduced in *ADH1B* KO as assessed by western blotting (Fig. 1*F* and *SI Appendix*, Fig. S3). Similarly, the protein levels of mature adipocyte markers, such as fatty acid synthase, perilipin1, adiponectin, and leptin, were also strikingly decreased in *ADH1B* KO cells (Fig. 1*F* and *SI Appendix*, Fig. S3).

### Rescue of Adipocyte Differentiation in *ADH1B*-Depleted ASC by 9-cis Retinoic Acid, but Not by Rosiglitazone.

**Fig. 2.**
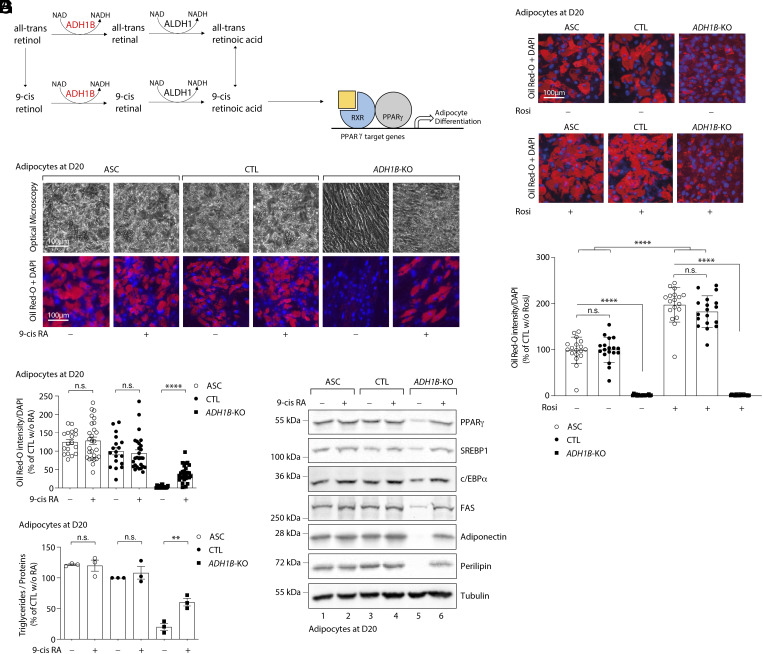
*ADH1B* deficiency can be bypassed by 9-cis RA treatment, but not by rosiglitazone. Data were obtained in ASC, ASC with a CRISPR-Cas9-mediated *ADH1B*-knockout (KO), and ASC transduced with a Cas9/scramble gRNA plasmid corresponding to control (CTL) cells. Adipocyte differentiation was induced in the presence or not of 9-cis RA (9-cis RA). Cells are studied at D20 postinduction. (*A*) Schematic representation of the link between ADH1B, 9-Cis RA, and adipocyte differentiation. The yellow square corresponds to 9-Cis RA. (*B*) Adipocyte differentiation assessed by Oil Red-O lipid staining. First line: representative pictures of cell dishes by optical microscopy. Second line: representative images of fluorescence microscopy after staining of intracellular lipids (Oil Red-O, red) and nuclei (DAPI, blue). Images are representative of three independent experiments. (*C* and *G*) Quantification of Oil Red-O fluorescence normalized to DNA content (DAPI) at D20. Results are expressed as means ± SEM of three independent experiments. (*D*) Intracellular triglyceride contents were measured at D20 in ASC, CTL, and *ADH1B* KO cells. The measurements are representative of three independent experiments. (*E*) Protein expression of adipocyte markers obtained by western blotting during in vitro adipocyte differentiation of ASC at D20. Numbers on the left correspond to molecular weight markers (kDa). Western blot images are representative of three independent experiments. PPARγ: peroxisome proliferator-activated receptor-gamma; C/EBPα: CCAAT/enhancer-binding protein-alpha; SREBP-1c: sterol regulatory element-binding protein-1c; FAS: fatty acid synthase. (*F*) Representative images of fluorescence microscopy after staining of intracellular lipids (Oil Red-O, red) and nuclei (DAPI, blue) in *ADH1B*-KO ASC. The first line depicts KO cells without rosiglitazone, while the second line shows those treated with it. (*G*) Quantification of Oil Red-O fluorescence normalized to DNA content (DAPI) at D20. Results are expressed as means ± SEM of three independent experiments. **P < 0.01, ****P < 0.0001, n.s.: nonsignificant.

When considering which specific product downstream of ADH1B might be responsible for its impact on adipocyte differentiation and function, we excluded ethanol metabolites, as ethanol is not a substrate to which all humans are universally exposed at meaningful levels. ADH1B is also highly active on retinol (vitamin A1), converting it to retinaldehyde (11–16). Retinaldehyde is then oxidized to several retinoic acid (RA) isomers (16), including 9-cis RA. Remarkably, 9-cis RA is the ligand for the retinoid X receptor (RXR), the obligate heterodimeric partner for PPARγ, which is the master regulator of adipogenesis (Fig. 2*A*). We speculated that ADH1B might be required to generate sufficient endogenous 9-cis RA to maintain human adipogenesis. To test this hypothesis, we treated *ADH1B* KO and CTL ASC with 9-cis RA at D0 and followed differentiation over 20 d. Treatment with 1 μM of 9-cis RA significantly improved adipogenic differentiation, lipid formation, and triglyceride accumulation in KO ASC (Fig. 2 *B*–*D*), whereas ASC and CTL cells were unaffected by this treatment (Fig. 2 *B*–*D*). Moreover, 9 cis-RA treatment of KO cells also restored the expression of adipogenic transcription factors, including PPARγ, C/EBPα, and SREBP1c (Fig. 2*E* and *SI Appendix*, Fig. S4), as well as mature adipocyte markers, including FAS, perilipin, and adiponectin (Fig. 2*E* and *SI Appendix*, Fig. S4).

To test whether a PPARγ agonist known to activate PPARγ-RXR heterodimers could also reverse the cell phenotype, we conducted adipocyte differentiation experiments with and without rosiglitazone. Rosiglitazone was not necessary to initiate the differentiation process, but significantly enhanced the differentiation of WT or CTL ASC (Fig. 2 *F* and *G*). In contrast, its addition to the culture medium had no effect on *ADH1B* KO cells and could not rescue the defect in adipocyte differentiation (Fig. 2 *F* and *G*).

### No Reversion of the Adipogenesis Block by Retinol or Low Doses of All-Trans RA.

**Fig. 3.**
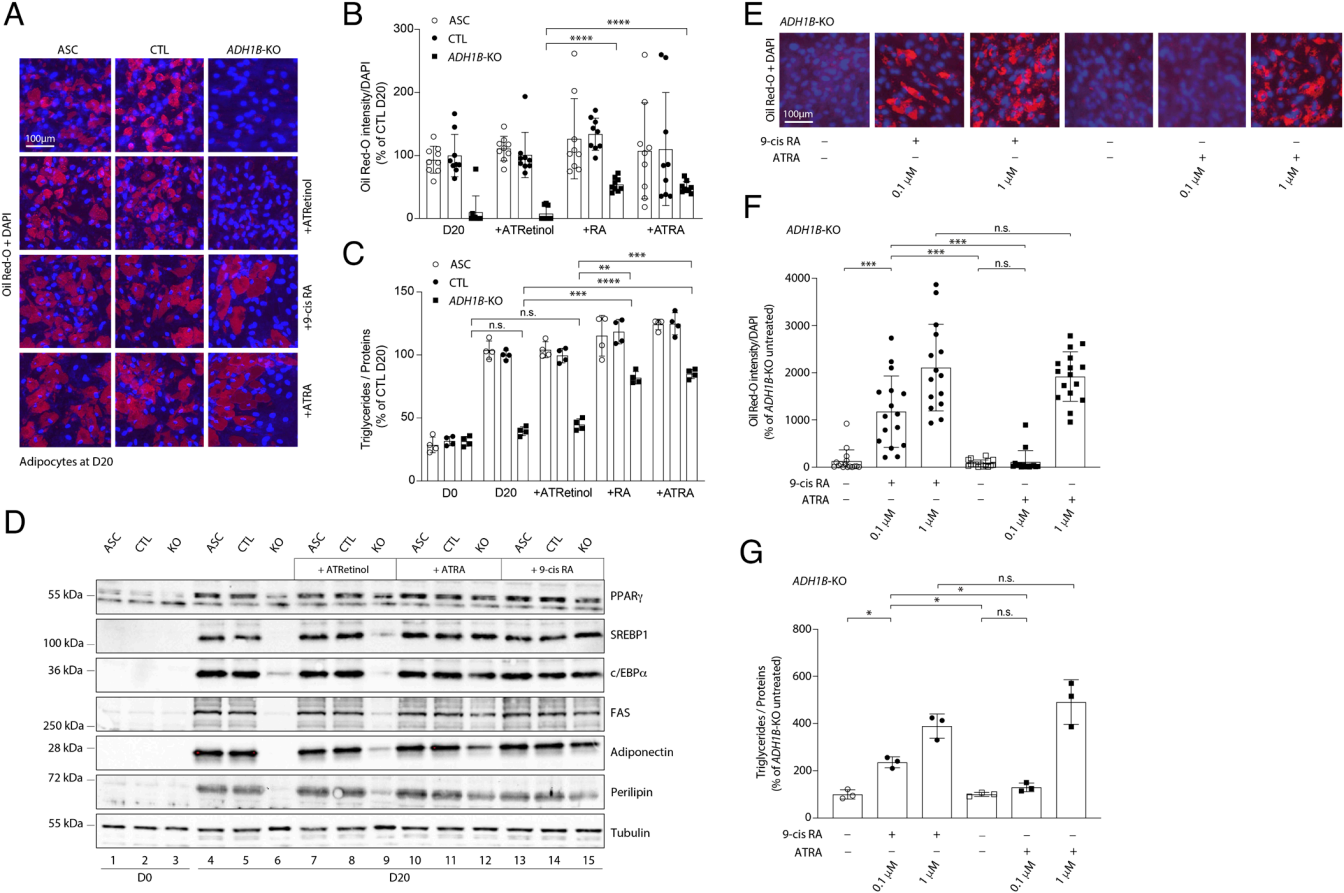
The block in adipogenesis is not rescued by retinol or small doses of ATRA. Data were obtained in ASC, ASC with a CRISPR-Cas9-mediated *ADH1B*-knockout (KO), and ASC transduced with a Cas9/scramble gRNA plasmid corresponding to control (CTL) cells. Adipocyte differentiation was induced in the presence or not of 9-cis RA, all-trans retinol (ATRetinol) and all-trans RA (ATRA). Cells are studied at D20 postinduction. (*A* and *E*) Adipocyte differentiation assessed by Oil Red-O lipid staining. Representative images of fluorescence microscopy after staining of intracellular lipids (Oil Red-O, red) and nuclei (DAPI, blue) are depicted. Images are representative of three independent experiments. (*B* and *F*) Quantification of Oil Red-O fluorescence normalized to DNA content (DAPI) at D20. Results are expressed as means ± SEM of three independent experiments. (*C* and *G*) Intracellular triglyceride contents were measured at D20 in ASC, CTL, and *ADH1B* KO cells. The measurements are representative of three independent experiments. (*D*) Protein expression of adipocyte markers obtained by western blotting during in vitro adipocyte differentiation of ASC at D20. Numbers on the left correspond to molecular weight markers (kDa). Western blot images are representative of three independent experiments. PPARγ: peroxisome proliferator-activated receptor-gamma; C/EBPα: CCAAT/enhancer-binding protein-alpha; SREBP-1c: sterol regulatory element-binding protein-1c; FAS: fatty acid synthase. **P < 0.01, ***P < 0.001, ****P < 0.0001.

In contrast to 9-cis RA, cell treatment with all-transretinol did not rescue the phenotype of *ADH1B* KO cells (Fig. 3 *A*–*D* and *SI Appendix*, Fig. S5). The fact adipogenesis in *ADH1B*-deficient ASC could be rescued by a product downstream of the enzyme but not by the corresponding substrate, strongly implicates ADH1B as an endogenous source of 9-cis RA that is required for human adipogenesis.

Surprisingly, adipogenesis was also rescued with all-trans RA (ATRA), which was unexpected considering that 9-cis RA is a more potent RXR activator than ATRA (Fig. 3 *A*–*D* and *SI Appendix*, Fig. S5) (17). Given that ATRA can isomerize at low rate to 9-cis RA (18), we hypothesized that part of the dose of ATRA used in our experiment might be converted to 9-cis RA at a rate sufficient to mediate a rescue effect. To test this hypothesis, we repeated the experiment with a 10-fold lower concentration of ATRA and 9-cis RA (0.1 μM), making ATRA conversion the limiting step in the process (Fig. 3 *E*–*G*). Our experimental data demonstrated that the low dose (0.1 μM) of 9-cis RA rescued adipocyte differentiation by 50% compared to the higher dose (1 μM). Most importantly, we found that this rescue effect was not observed with the lower dose of ATRA (0.1 μM) (Fig. 3 *E*–*G*). This suggests that 9-cis RA is more potent than ATRA at rescuing adipocyte differentiation at this lower dose, strongly indicating that the rescue effect is mediated by 9-cis RA, the known ligand for RXR.

### Identification of an *ADH1B* Variant Affecting Enzyme Homodimerization and Activity in a Patient with Lipodystrophy.

**Fig. 4.**
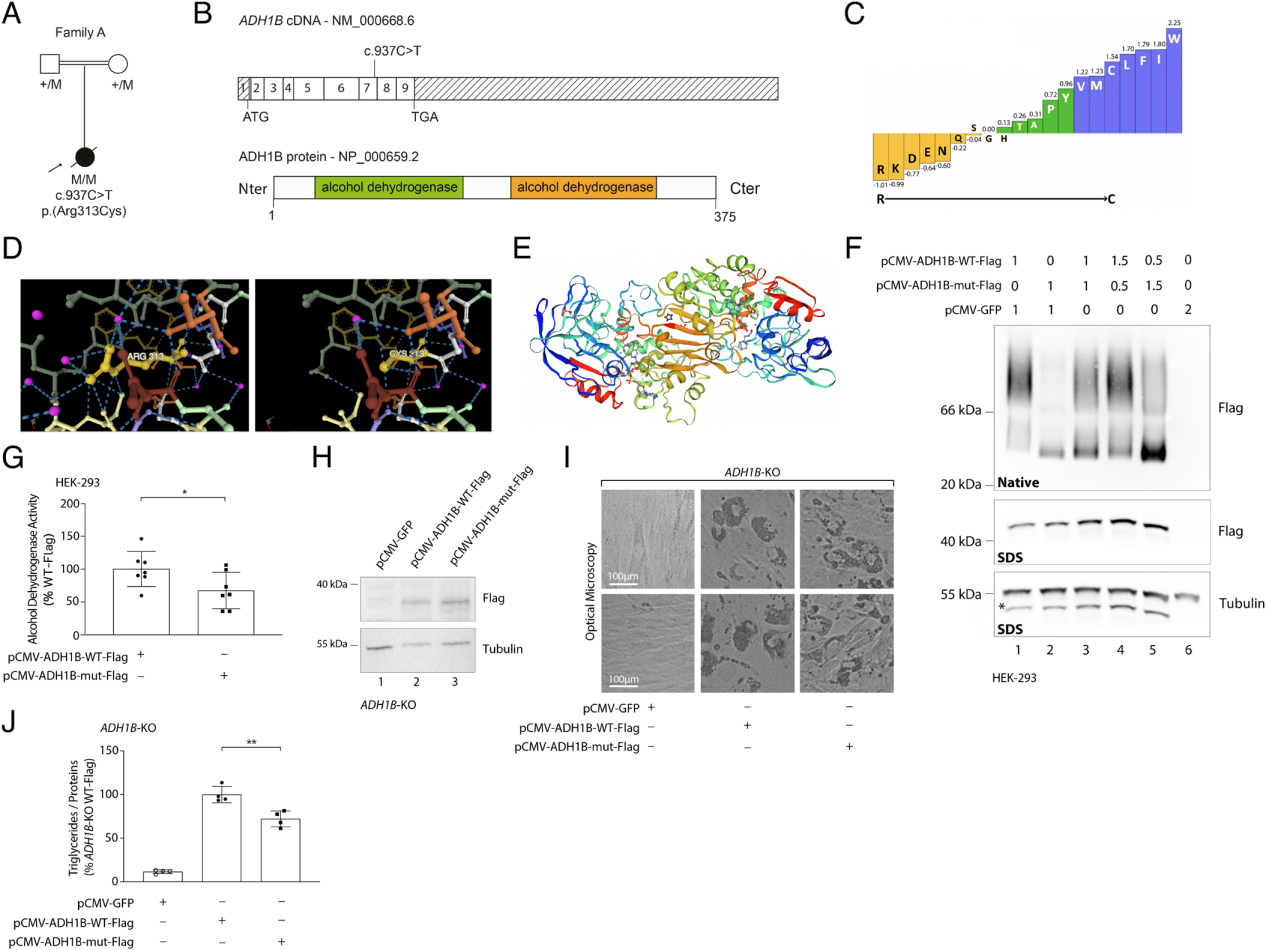
Identification of an *ADH1B* homozygous variant affecting ADH1B homodimerization and enzyme activity in a patient with generalized lipoatrophy. (*A*) Genealogical tree and segregation analysis for the *ADH1B* variant in the family investigated herein. The arrow indicates the proband. +, normal allele; M, mutant allele. (*B*) *Top* panel: schematic representation of *ADH1B* transcript sequence (NM_000668.6) displaying the location of the variant identified. *Bottom* panel: schematic representation of ADH1B protein sequence comprising 375 amino acids. The prediction of protein domain organization was based on the UniProt database (protein reference: P00325). (*C*) Classification of amino acids in order of increasing hydrophobicity using a Fauchère and Pliska plot and showing the major change induced by the p.Arg313Cys variant. (*D*) Prediction of the effect of p.Arg313Cys variant on ADH1B 3D structure. This was performed using the MIZTLI software (https://miztli.biokerden.eu/) and the 1u3u ADH1B structure from Protein Data Bank (https://www.wwpdb.org/). Arg313 was replaced by a cysteine residue using FASPR (23). Dashed lines represent noncovalent hydrogen bonds. The p.Arg313Cys variant induces the loss of a number noncovalent interactions with other ADH1B residues, which is predicted to affect the protein stability. (*E*) Model of the 3D structure of ADH1B extracted from SWISS MODEL repository and corresponding to the crystal structure of a human homodimer of ADH1B (https://swissmodel.expasy.org—protein reference: P00325). The location of the p.Arg313Cys variant identified in patient 1 is indicated by a star. (*F*) Transfection of HEK 293 cells with plasmids encoding wild-type (WT) ADH1B and the p.Arg313Cys mutated form of the protein with a C-terminal Flag tag. Cellular extracts were subjected to native-PAGE (*Upper* panel) vs. SDS-PAGE (*Middle* panel) to evaluate the impact of the variant on ADH1B homodimerization. *: The lowest band on the gel corresponds to the one obtained with the anti-Flag antibody, since the same membrane was blotted to reveal tubulin. (*G*) Assessment of ADH activity in HEK 293 cells stably expressing either WT or mutant forms of ADH1B. The measurements are representative of three independent experiments. (*H*) WB analysis performed on *ADH1B* KO cells nucleofected with plasmids encoding ADH1B WT, mutant, or green fluorescent protein (GFP). (*I*) Optical microscopy images captured from *ADH1B* KO cells complemented with ADH1B WT, mutant, or GFP at D15 after adipocyte differentiation induction. (*J*) Measurement of intracellular triglyceride levels at D15 in *ADH1B* KO cells complemented with ADH1B WT, mutant, or GFP as a negative control. The measurements are representative of two independent experiments in duplicates. *P < 0.05, **P < 0.01.

The fact that human adipocyte differentiation appears to require *ADH1B* makes this an obvious candidate gene for lipodystrophy. We studied a cohort of 263 patients with lipodystrophy syndromes, referred for genetic testing to the Assistance Publique-Hôpitaux de Paris (AP-HP) institute, and carrying no pathogenic variant in a known causative gene (19). These patients were screened for molecular defects in *ADH1B* by means of a gene panel. One patient, an Egyptian female, referred to as Patient 1, was found to carry a homozygous missense variant in the exon 7 of *ADH1B*: c.937C>T (NM_000668.6); p.Arg313Cys (Fig. 4 *A* and *B*). Patient 1 presented in early childhood with generalized lipoatrophy, insulin-resistant diabetes, liver steatosis which progressed to fibrosis, leading to death at the age of 13 y. The variant was present in the heterozygous state in her parents, who were first cousins and asymptomatic. Although this variant is very rare in the general population with a minor allele frequency (MAF) in gnomAD of 5.10^−5^, it is found more frequently in the Middle East population (MAF = 0.01 in 314 individuals − gnomAD v3.1.2). We then examined the Qatar Biobank (n = 3,000 individuals) and found a MAF of 0.03, including one homozygote, who was reportedly healthy. Although this variant is clearly not the singular cause of the lipodystrophy found in Patient 1, we wondered whether it could predispose to adipocyte dysfunction under some circumstances. This variant replaces arginine which has a large positively charged side chain with cysteine, which has a small nonpolar side chain (Fig. 4*C*). Inspection of the known quaternary structure of the ADH1B dimer (2, 20, 21), suggested that the variant would result in loss of a number of hydrogen bonds with other ADH1B residues within the same monomer (Fig. 4*D*). Using a 3D structure model from the SWISS-MODEL repository (22), we determined that Arg313 is located at the interface between the two monomeric subunits (Fig. 4*E*).

To assess the impact on ADH1B dimerization, HEK 293 cells were transfected with vectors expressing WT and mutated forms of ADH1B. While western blotting revealed that ADH1B could form homodimers, the p.Arg313Cys mutated form of ADH1B appeared almost exclusively as a monomer, confirming the in silico predictions (Fig. 4*F*). Coexpression of the WT and mutated forms showed a dose-dependent inhibition of dimer formation by the mutant (Fig. 4*F*), indicating a loss of function associated with the variant. Enzyme activity measurement in HEK293 cells using a commercial colorimetric assay demonstrated a 25% loss of activity with the p.Arg313Cys variant, consistent with the loss of dimer formation (Fig. 4 *F* and *G*). Furthermore, reexpression of WT and mutated forms of ADH1B in *ADH1B* KO cells partially restored adipocyte differentiation, with a more significant improvement observed with the WT form, as evidenced by increased triglyceride levels (Fig. 4 *H–J*). This confirmed that the defect in adipogenesis observed in KO cells was due to the loss of the enzyme, and supported a loss-of-function effect of the variant.

## Discussion

ADH1B is mainly known for its role in ethanol catabolism (5–7), and several *ADH1B* single nucleotide polymorphisms have been associated with the risk of alcohol consumption and dependence (24, 25). The contribution of mouse models to understanding the physiological role of ADH in humans has been somewhat limited, since mice only have five *Adh* genes and none of them has the same expression pattern as human *ADH1B*, with its high expression in adipose tissue as well as liver.

Previous studies have indicated that, in human adipocytes, *ADH1B* expression is influenced by metabolic state (8–10), and that knock down of its expression reduces the extent of adipocyte differentiation (9, 10). In our work, we have used CRISPR-Cas9 technology to completely or near completely remove ADH1B expression from human ASC and found that this profoundly impairs adipocyte differentiation. The fact that retinol is a known substrate for ADH1B along with the well-known role of 9-cis RA as the ligand for RXR (16), the obligate heterodimeric partner of PPARγ, led us to perform rescue experiments using RA and retinol. We demonstrated that treatment of *ADH1B*-KO ASC with 9-cis RA, but not with all-transretinol, substantially rescues adipocyte differentiation. These findings indicate that ADH1B acts as an essential source of 9-cis RA required to support human adipogenesis. It might seem odd that the rescue in adipocyte differentiation with 9-cis RA is only partial. It is possible that transport of 9 cis-RA into the adipocyte is somehow rate limiting and that we cannot fully compensate for a defect in intra-adipocyte generation of 9 cis-RA by supplying it extracellularly. It is also possible that intracellularly generated 9 cis-RA is more effectively channeled to the nucleus.

The fact that ADH1B enzymatic activity appears critical for human adipogenesis raises the possibility that genetic variation affecting the enzyme could predispose to human metabolic disease. The most extreme form of adipose dysfunction is congenital generalized lipodystrophy. In one such patient (with no pathogenic variant in the genes known to cause lipodystrophy), we found a homozygous missense variant which significantly impairs dimerization of the ADH1B monomers and alters the enzyme activity. The Arg313Cys variant appears to be enriched in Middle East populations and, among 3,000 Qatari biobank participants, one healthy male was homozygous for the same variant. Thus, it is very unlikely that the Arg313Cys, acting alone, is responsible for the severe congenital lipodystrophy seen in the Egyptian proband. However, it is possible that it might contribute to adipocyte dysfunction depending on other background genetic and environmental factors. It is difficult to assess whether more subtle genetic variations affecting *ADH1B* have an impact on fat cells more generally in humans, as impacts of such variations on measures of adiposity are highly confounded by substantial effects of variants in this gene on ethanol tolerance and consumption (24, 25).

Many questions remain to be answered. Future studies will establish whether the altered lipid composition that results from loss of ADH1B is correctable by provision of 9-cis RA. RXR is ubiquitously expressed and essential for numerous developmental and physiological functions. Why is it only in adipocytes that an endogenous source of its ligand is essential and why only in humans? Importantly, while the results of our experiments ablating ADH1B in human ASC are consistent with previous work where ADH1B expression was knocked down, to date, there is no evidence that this happens in vivo. The increasing availability of large-scale human exome and genome sequencing data combined with links to phenotype should, in time, provide information about the in vivo consequences of *ADH1B* deficiency. It is conceivable that ethanol could compete with retinol for metabolism in adipocytes. Chronic severe alcohol excess is frequently accompanied by a “Cushingoid” appearance with reduction in subcutaneous fat and increase in visceral fat (26). Future studies should examine the interaction of ethanol and retinol in human adipocytes and whether there are any regional differences in adipocyte ADH1B’s susceptibility to substrate competition.

In summary, our studies have revealed that a classical member of the class 1 ADH family has an unexpected, cell-autonomous role in providing RA to facilitate human adipogenesis.

## Materials and methods

**Key resources table**

**Table:** 

REAGENT or RESOURCE	SOURCE	IDENTIFIER
Antibodies		
Anti-ADH1B	Protein Tech	Cat# 17165-1-AP
Anti-Adiponectin	Thermo Fisher Scientific	Cat# MA1-054
Anti-C/EBPα	Protein Tech	Cat# 18311-1-AP
Anti-FAS	Cell Signaling Technology	Cat# 3180
Anti-Leptin	Thermo Fisher Scientific	Cat# PA1-051
Anti-Perilipin-1	Abcam	Cat# ab3526
Anti-PPARγ	Protein Tech	Cat# 16643-1-AP
Anti-SREBP-1	Santa Cruz Biotechnology	Cat# sc-366
Anti-Tubulin	Sigma-Aldrich	Cat# T5168
Anti-rabbit-HRP	Cell Signaling Technology	Cat# 7074
Anti-mouse-HRP	Cell Signaling Technology	Cat# 7076
Biological Samples		
Adipose Stem Cells (ASC)	Pr. Fève Lab at CRSA, Paris	N/A
Fetal calf serum	Sigma-Aldrich	Cat# F7524
Newborn calf serum	Biosera	Cat# CA-1151500
Chemicals, Peptides, and Recombinant Proteins		
2,7-dichlorodihydrofluorescein diacetate (CM-H_2_DCFHDA)	Sigma-Aldrich	Cat# C6827
3-isobutyl-1-methyl xanthine (IBMX)	Sigma-Aldrich	Cat# I7018
9-cis-retinoic acid (CAS 5300-03-8)	Santa Cruz Biotechnology	Cat# sc-205589
All-trans-retinoic acid (CAS 302-79-4)	Sigma-Aldrich	Cat# R2625
All-trans-retinol (CAS 68-26-8)	Sigma-Aldrich	Cat# R7632
DAPI	Sigma-Aldrich	Cat# D1306
Dexamethasone	Sigma-Aldrich	Cat# D4902
Fibrobast Growth Factor-2 (FGF-2)	PeproTech	Cat# 100-18B
GlutaMAX	Thermo Fisher Scientific	Cat# 35050061
G-418 Sulfate	Sigma-Aldrich	Cat# G418-RO
HEPES	Thermo Fisher Scientific	Cat# 15630056
Insulin	Sigma-Aldrich	Cat# I0516
Oil Red-O	Sigma-Aldrich	Cat# O0625
Paraformaldehyde	Thermo Fisher Scientific	Cat# J19943.K2
Penicillin/streptomycin	Thermo Fisher Scientific	Cat# 11548876
Rosiglitazone	Sigma-Aldrich	Cat# D2408
Critical Commercial Assays		
Human MSC Kit buffer	Lonza	Cat# VPE-1001
MycoAlert^TM^ PLUS Mycoplasma Detection Kit	Lonza	Cat# LT07-701
QuantiChrom^TM^ Alcohol dehydrogenase Assay Kit	BioAssay Systems	Cat# DADH-100
QuikChange II Site-directed mutagenesis kit	Agilent Technologies	Cat# 200523
Infinity Kit™	Thermo Fisher Scientific	Cat# TR22421
TurboFect™	Thermo Fisher Scientific	Cat# R0532
Recombinant DNA		
lentiCRISPR v2	Addgene	Cat# 52961
pCMV6-entry-GFP	Origene	Cat# PS100026
pCMV-ADH1B WT-Flag	Origene	Cat# RC205391L3
pCMV-ADH1B c.937C>T-Flag	Described in Methods	N/A
Software and Algorithms		
Multi-Experiment Viewer (MeV)	WebMev	N/A
Prism	GraphPad Software	N/A
Web resources		
CADD	https://cadd.gs.washington.edu	
CRISPOR	http://crispor.tefor.net/	
GnomAD	https://gnomad.broadinstitute.org	
GTEx	https://gtexportal.org/home	
MIZTLI	https://miztli.biokerden.eu/	
PolyPhen-2	http://genetics.bwh.harvard.edu/pph2	
Protein Data Bank	https://www.wwpdb.org/	
SIFT	https://sift.bii.a-star.edu.sg/	
SWISS-MODEL	https://swissmodel.expasy.org	

### Materials Availability.

Unique materials generated in this study are available upon complete materials transfer agreement.

### Study Approval.

Written informed patient consent was obtained for the genetic study. The study was approved by the CPP Ile de France 5 research ethics board (DC 2009-963, Paris, France).

### ASC Isolation, Culture, and Adipocyte Differentiation.

ASC isolation and differentiation procedures followed the protocols outlined in previous studies (27–29). In brief, ASC were obtained from surgical samples of subcutaneous abdominal adipose tissue sourced from a 25-y-old healthy woman with a normal body mass index (BMI). Adipose tissue underwent enzymatic digestion using collagenase B (0.2%). Following centrifugation, stromal vascular fraction was filtered, washed, plated, and cultured in α-MEM (#12571063; Thermo Fisher Scientific, MA) supplemented with 10% fetal calf serum (FCS, #F7524; Sigma-Aldrich, MO), 1% GlutaMAX (#35050061; Thermo Fisher Scientific), 1% penicillin/streptomycin (P/S – 10,000 UI/mL, #11548876; Thermo Fisher Scientific), 1% HEPES (#15630056; Thermo Fisher Scientific), and fibroblast growth factor-2 (FGF-2 – 145 nmol/L, #100-18B; PeproTech, Neuilly-sur-Seine, Paris). After 24 h, only ASC adhered to plastic surfaces, while other cells were removed during medium replacement. ASC were maintained in an undifferentiated state in α-MEM supplemented with 10 % newborn calf serum (#CA-1151500; Biosera, MI), 1% GlutaMAX, HEPES and P/S, and FGF-2 (145 nmol/L) and routinely screened for mycoplasma contamination (MycoAlert^TM^ PLUS Mycoplasma Detection Kit; #LT07-701; Lonza, Bale, Switzerland). Adipocyte differentiation was induced by treating 2-d postconfluent cultures with high-glucose (25 mmol/L) Dulbecco’s Modified Eagle’s Medium (DMEM, #11960085; Thermo Fisher Scientific) supplemented with 10 % FCS, 1 % P/S, 1 µmol/L dexamethasone (#D4902; Sigma-Aldrich), 1 µmol/L rosiglitazone (#D4902; Sigma-Aldrich), 250 µmol/L 3-isobutyl-1-methyl xanthine (IBMX) (#I7018; Sigma-Aldrich), and 0.17 µmol/L insulin (#I0516; Sigma-Aldrich) for a duration of 10 d. The medium was then replaced with high-glucose DMEM supplemented with 10% FCS, 1 % P/S, 1 µmol/L rosiglitazone, and 0.17 µmol/L insulin, with subsequent medium changes every 2 d until the 20th day. For rescue experiments, 9-cis RA (#sc-205589; Santa-Cruz, TX), all-trans retinol (#R7632; Sigma-Aldrich), and all-trans RA (#R2625; Sigma-Aldrich) were added to the differentiation cocktail at 1 or 0.1 µmol/L.

### CRISPR/Cas9-Mediated Deletion of *ADH1B*.

CRISPR/Cas9-mediated deletion of *ADH1B* was conducted using the lentiviral plasmid plentiCRISPRv2, generously provided by the Zhang lab (Addgene, MA, USA; plasmid #52961), which contains hSpCas9, a guide RNA (gRNA), and a puromycin resistance sequence. The gRNA designed to target exon 4 of *ADH1B* was meticulously selected using the established tool (http://cistrome.org/SSC) to ensure both specificity and high cleavage efficiency. To mitigate off-target effects, the web-based tool CRISPOR (http://crispor.tefor.net) (30) was used to exclude potential off-target sequences (*SI Appendix*, Table S1). Lentiviruses tailored for *ADH1B* knockdown were generated by the VVTG platform (Federative Research Institute, Necker, France). ASC transduction followed established protocols (27–29), wherein viral particles were introduced to ASC at a minimal titer of 10^8^ units per mL. Subsequently, transduced cells were selected with 0.5 μg/mL puromycin dihydrochloride (#P9620; Sigma-Aldrich) 48 h postinfection. Surviving cells were expanded, and the resulting heterogeneous cell pool was used for subsequent experiments. Evaluation of on-target recombination, including insertions and deletions (indels), in the genomic DNA from this cell population was performed via Sanger sequencing of *ADH1B* exon 4, followed by analysis using the Synthego web-based tool (https://ice.synthego.com) (*SI Appendix*, Figs. S1 and S2). The gRNA sequence used in this study was the following:

**Table:** 

*gRNA*	*Sequence*
*ADH1B*	5′-CCGCTCTTTACTCCTCAGTG-3′

### Western Blot.

Western blot analysis was conducted following established protocols detailed in prior publications (27–29). Cells were lysed in NP-40 lysis buffer, and protein extracts containing 30 μg of protein were separated by sodium dodecyl sulfate–polyacrylamide gel electrophoresis (SDS-PAGE), before being transferred to polyvinylidene difluoride membrane for immunoblotting using appropriate antibodies (refer to the detailed list below). Quantification of western blot was performed in triplicate using Fiji software (open source), with normalization to the tubulin protein levels. Uncropped and unedited western blots corresponding to the figures presented are available in *SI Appendix*.

### Homodimerization and ADH Assays.

Human embryonic kidney HEK-293 (ATCC®-CRL-1573^TM^) cells were cultured in high-glucose (4.5 g/L) DMEM (Thermo Fisher Scientific) containing 10% fetal calf serum and 1 % P/S. The pCMV6-entry mammalian expression vector containing the coding sequence for *ADH1B* with a C-terminal Flag Tag was purchased from Origene (#RC205391L3; Origene, MD). The *ADH1B* c.937C>T variant was introduced using the QuikChange II Site-directed mutagenesis kit (#200523; Agilent Technologies, CA), and constructs were checked by Sanger sequencing. For the dimerization assay, transient transfection of the different cell lines was carried out in six-well plates with TurboFect™ Transfection Reagent (#R0532; Thermo Fisher Scientific) according to the manufacturer’s instructions. The NativePAGE™ Novex Bis-Tris Gel system was used to perform native (nondenaturing) electrophoresis, according to the manufacturer’s instructions. For the ADH assay, stable expression of WT or mutant ADH1B in HEK-293 cells was achieved by selection with 400 μg/mL of G-418 sulfate (#G418-RO; Sigma-Aldrich) for three weeks, followed by maintenance in the presence of 100 μg/mL of G-418 sulfate. ADH activity was determined using the Quantitative Colorimetric Kinetic ADH Activity Kit (#DADH001; BioAssay Systems, CA) following the manufacturer’s instructions.

### Nucleofection Procedure.

*ADH1B* KO ASC were cultured until they reached 80% confluency, and the medium was changed the day before nucleofection. For each nucleofection assay, 6 × 10^5^ cells were resuspended in 100 μL of nucleofector buffer (Human MSC [mesenchymal stem cells] Kit buffer, reference VPE-1001; Lonza, Basel, Switzerland) and nucleofected with 2 μg of plasmid DNA. The C-17 protocol was employed using the Nucleofector II device. Immediately after nucleofection completion, cells were seeded onto 24 mm dishes. The culture medium was changed 6 h postnucleofection to promptly remove dead cells. Expression was assessed via western blotting 36 h after nucleofection, and adipocyte differentiation was initiated.

### Oil Red-O Staining, Image Processing, and Quantification.

Intracellular lipid content was visualized using Oil Red-O staining (#O0625; Sigma-Aldrich), following established procedures (27–29). Cells were rinsed with phosphate-buffered saline (PBS) and fixed with 4 % paraformaldehyde (PFA, #J19943.K2, Thermo Fischer Scientific) in PBS for 10 min. Subsequently, fixed cells were incubated with Oil Red-O solution for 1 h at room temperature, followed by incubation with DAPI (#D1306, Sigma-Aldrich) for 5 min. Fluorescence imaging was conducted using an IX83 Olympus microscope, with image acquisition facilitated by Cell-Sens V1.6 software and subsequent analysis carried out using Fiji software. Images of 8 to 10 different areas per condition were captured via fluorescence microscopy using the mCherry filter. Subsequent image processing was conducted through Fiji software. Specifically, images were subjected to threshold conversion, converting the 8-bit Red-Green-Blue image into a binary image, which consists only of pixels representing lipid droplets (i.e., red). It is noteworthy that the resulting binary image was meticulously cross-verified with the original image to ensure consistency and accurate binary conversion. The surface area occupied by lipid droplets within the image was quantified by Fiji software in μm^2^ and normalized to cell number through semiautomated counting of DAPI-stained nuclei.

### Quantification of Intracellular Triglyceride Content.

Quantification of intracellular triglyceride content followed the methodologies outlined in prior studies (28, 29). Intracellular lipids from differentiated ASC were extracted using hexane/isopropyl alcohol (3:2). Cells were washed and then incubated with hexane/isopropyl alcohol (3:2, vol/vol) using 500 µL per well in 6-well culture plates, placed on a shaker (80 rpm/min) at room temperature for 60 min. Subsequently, the contents of each well were transferred into glass tubes for nitrogen evaporation of the organic solvent. After evaporation, lipids were resuspended in isopropyl alcohol and transferred in duplicate into 96-well plates for analysis following drying. Triglycerides were quantified using the Infinity™ Triglyceride kit (#TR22421; Thermo Fischer Scientific) according to the manufacturer’s instructions. The absorbance of each well was measured using a Tecan microplate reader (TECAN, Männedorf, Switzerland) and converted to concentration based on a standard curve. Results were then normalized to the cell protein content.

### Genetic Analyses.

#### *Gene panel*.

Genomic DNA extracted from peripheral blood leukocytes underwent analysis using a panel consisting of the following genes: *ADH1B*, *ADRA2A*, *AGPAT2*, *AIRE*, *AKT2*, *BANF1*, *BLM*, *BSCL2*, *CAV1*, *CAVIN1*, *CIDEC*, *DYRK1B*, *EPHX1*, *ERCC3*, *ERCC6*, *ERCC8*, *FBN1*, *INSR*, *LEMD2*, *LIPE*, *LMF1*, *LMNA*, *LMNB2*, *MDM2*, *MFN2*, *MTX2*, *NSMCE2*, *PCNT*, *PCYT1A*, *PIK3R1*, *PLIN1*, *POC1A*, *POLD1*, *POLR3A*, *PPARG*, *PTPN11*, *POMP*, *PSMA3*, *PSMB4*, *PSMB8*, *PSMB9*, *PSMG2*, *OTULIN*, *SLC29A3*, *SPRTN*, *WRN*, and *ZMPSTE24*. Exons along with flanking intronic sequences were captured from fragmented DNA with the SeqCapEZ enrichment protocol (Roche NimbleGen, WI, USA), followed by paired-end massively parallel sequencing on a MiSeq platform (Illumina, CA) (19). Bioinformatic analysis of the sequencing data was carried out using the Sophia DDM pipeline^®^ (Sophia Genetics, Switzerland).

#### *Sanger sequencing*.

PCR amplification was performed, followed by sequencing using the Big Dye Terminator v3.1 sequencing kit (Thermo Fisher Scientific, MS, USA). Data analysis was conducted on a 3500xL Dx device using SeqScape v2.7 software (Thermo Fisher Scientific).

### Statistical Analysis.

Data are presented as means ± SD (SE). *P* < 0.05 was considered statistically significant. For cellular biology studies, GraphPad Prism software (California) was used to evaluate statistical significance. Gaussian distribution was tested with the D’Agostino–Pearson test. Multiple comparisons were conducted by one-way ANOVA with Bonferroni test or Kruskal–Wallis test for post hoc analysis.

## Supplementary Material

Appendix 01 (PDF)

Appendix 02 (PDF)

Dataset S01 (DOCX)

## Data Availability

All study data are included in the article and/or supporting information.
