# Supplementary material for: ADH1B, the adipocyte-enriched alcohol dehydrogenase, plays an essential, cell-autonomous role in human adipogenesis
Source: Proc Natl Acad Sci U S A. 2024 Jun 5;121(24):e2319301121. doi: 10.1073/pnas.2319301121 (PMC11181076; doi:10.1073/pnas.2319301121)
Supplement: Supplementary file 1 — Appendix 01 (PDF) [file pnas.2319301121.sapp01.pdf]

PNAS

Supporting Information for
ADH1B, the adipocyte-enriched alcohol dehydrogenase, plays an essential, cell-autonomous role in human adipogenesis

Jérémie Gautheron[1,2#], Solaf Elsayed[3], Valeria Pistorio[1,2], Sam Lockhart[4], Jamila Zammouri[1,2], Martine Auclair[1,2], Albert Koulman[4], Sarah R Meadows[4], Marie Lhomme[5], Maharajah Ponnaiah[6], Redouane Si-Bouazza[7], Sylvie Fabrega[7], Abdelaziz Belkadi[8], Qatar Genome Project[9], Jean-Louis Delaunay[1,2], Tounsia Aït-Slimane[1,2], Bruno Fève[1,2,10], Corinne Vigouroux[1,2,10], Tawhida Y Abdel Ghaffar[11], Stephen O'Rahilly[4,13#], Isabelle Jéru[1,2,12,13#].

#Co-corresponding authors

Jérémie Gautheron
Email: jeremie.gautheron@inserm.fr

Isabelle Jéru
Email: isabelle.jeru@aphp.fr

Stephen O'Rahilly
Email: so104@cam.ac.uk

This PDF file includes:

Figures S1 to S5
Table S1

Other supporting materials for this manuscript include the following:

Datasets S1

Group Author Information

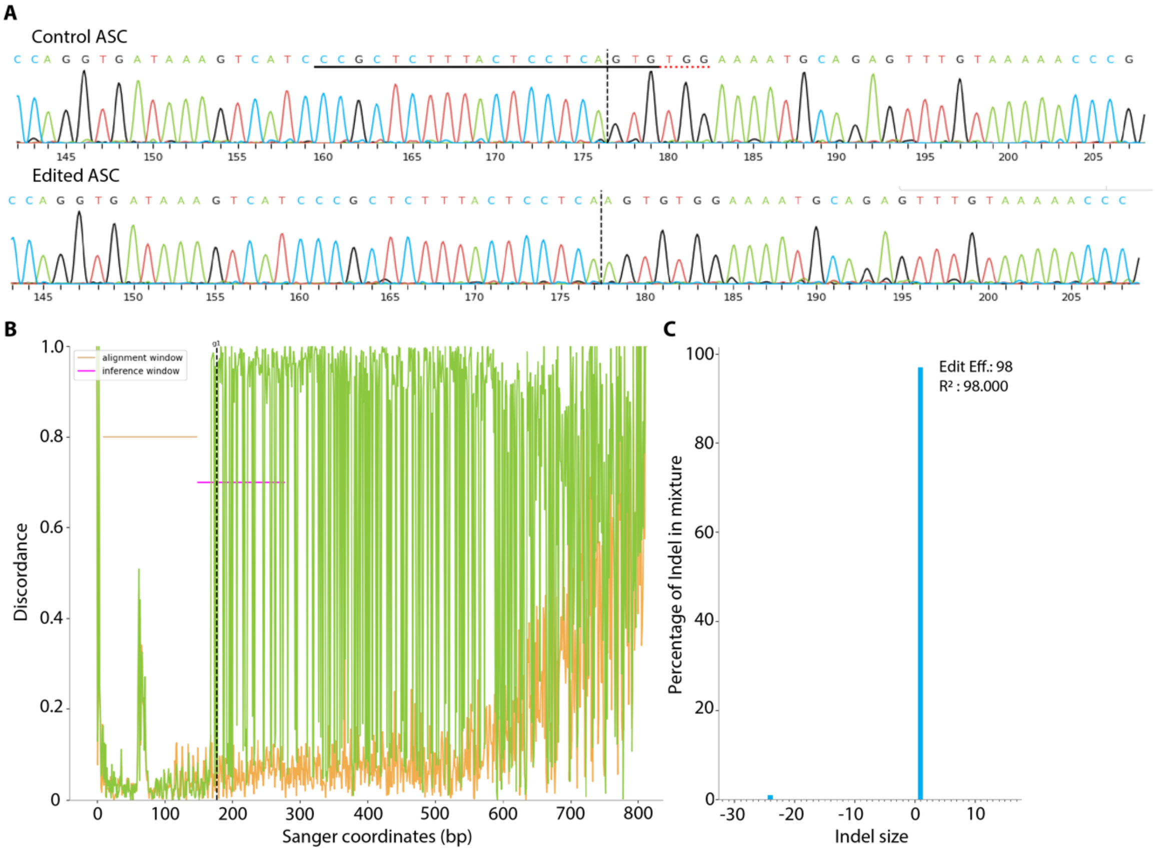

Fig. S1. Assessment of CRISPR/Cas9 editing efficiency with gRNA targeting *ADH1B* exon 4 in control and edited ASC. (A) Sanger sequencing of the target region (exon 4) confirmed high level of recombination. The gRNA sequence is underlined. (B) Determination of CRISPR indel pattern in control and edited ASC by analyzing Sanger sequencing data with the Tide software (https://tide.nki.nl). The discordance plot details the rate of sequence alignment per base between the control and edited samples in the inference window (*i.e.*, the region around the recombination site highlighted by a vertical dotted blue line and the gRNA sequence is underlined). Before the gRNA target site, the green line (edited sample) and the orange line (control sample) are close together. After the gRNA target site, a jump is observed, corresponding to a high level of sequence misalignment. (C) The graph displays the frequency of indels in relationship with the indel size. The editing efficiency corresponding to the recombination rate was evaluated at 98%.

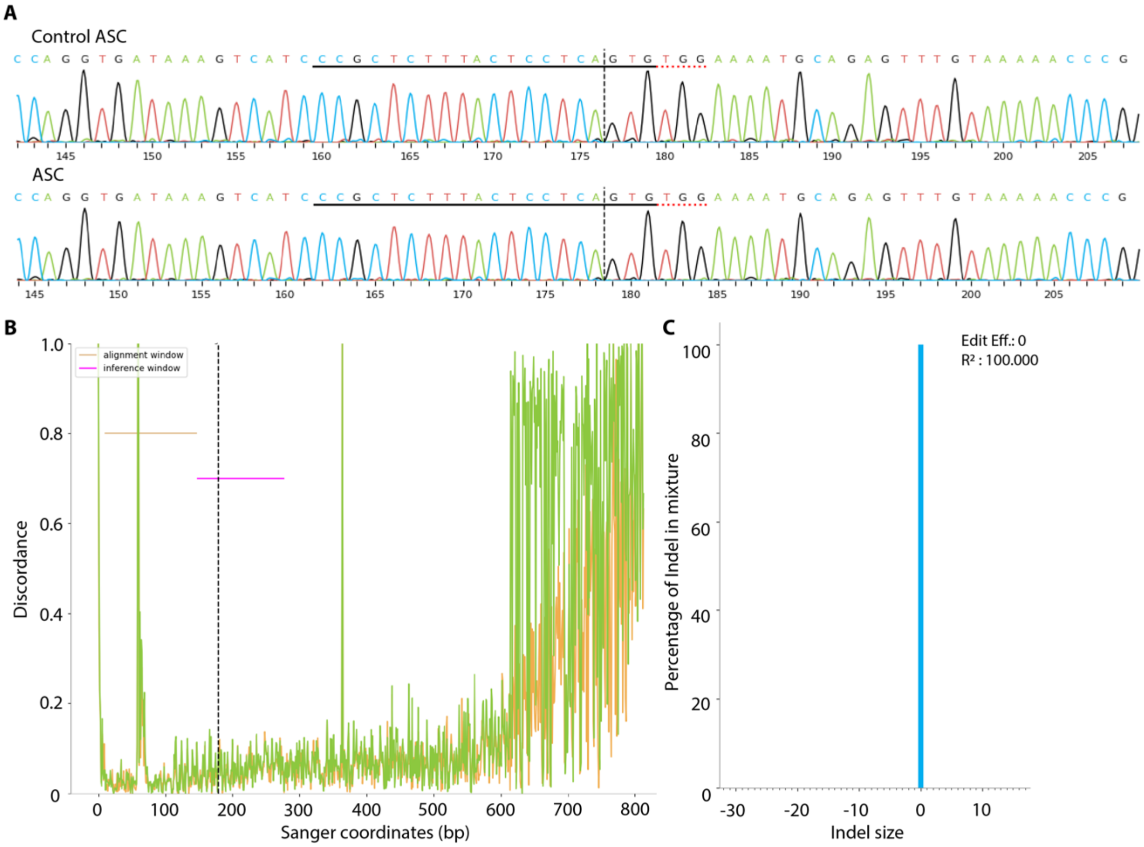

Fig. S2. Assessment of CRISPR/Cas9 editing efficiency with gRNA targeting *ADH1B* exon 4 in edited control and ASC. (A) Sanger sequencing of the target region (exon 4) confirmed an absence of recombination. The gRNA sequence is underlined. (B) Determination of CRISPR indel pattern in edited control and ASC by analyzing Sanger sequencing data with the Tide software (https://tide.nki.nl). (C) The graph displays the frequency of indels in relationship with the indel size. The recombination rate was evaluated at 0%.

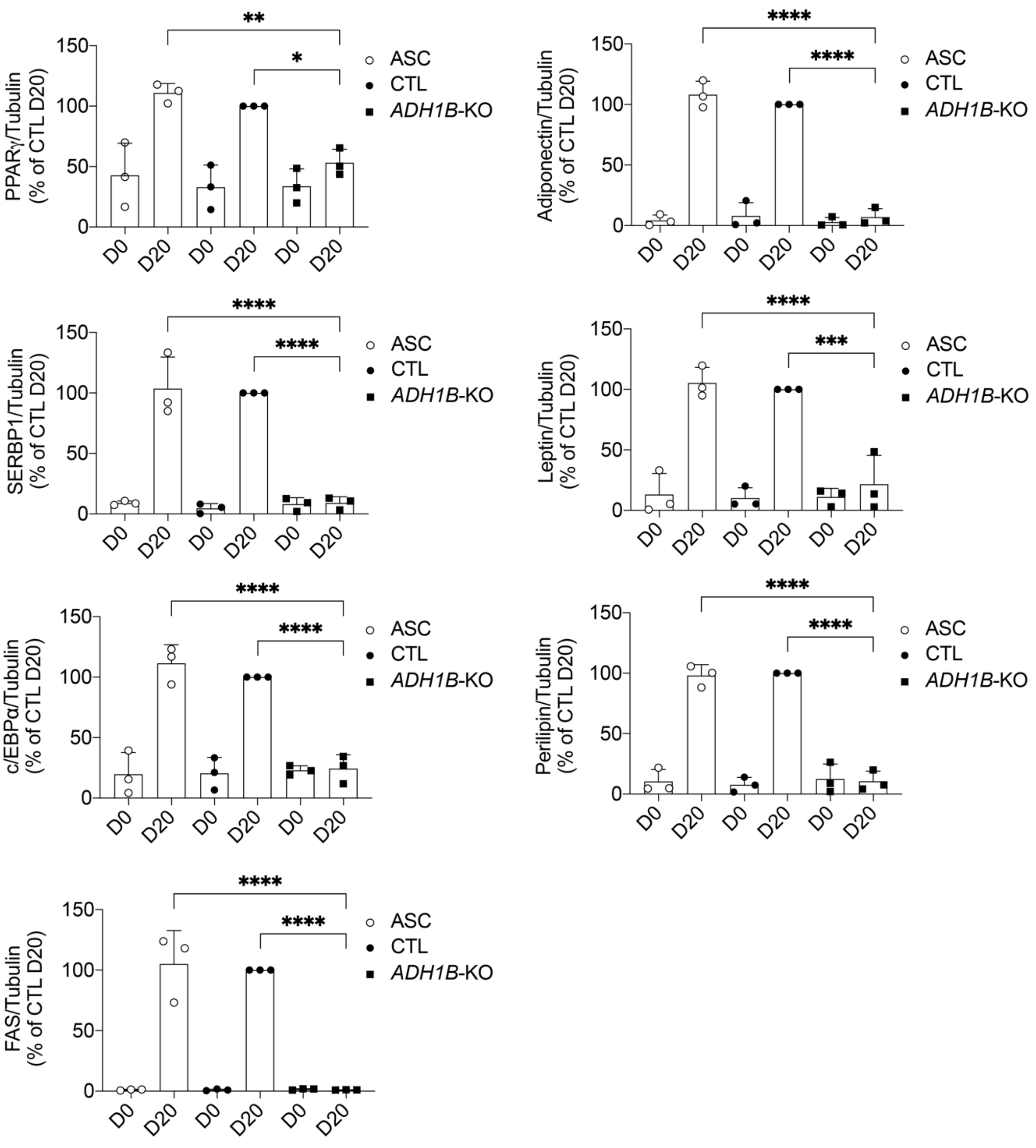

Fig. S3. Evaluation of adipocyte markers in control and edited ASC. Data were obtained in ASC, ASC with a CRISPR-Cas9-mediated *ADH1B* knockout (KO), and ASC transduced with a Cas9/scramble gRNA plasmid corresponding to control (CTL) cells. The Western blots of adipocyte markers related to Figure 2F were quantified using FIJI software. *p*-values were determined by analysis of variance (ANOVA) with Bonferroni's *post hoc* multiple comparison test. *$p < 0.05$, **$p < 0.01$, ***$p < 0.001$ and ****$p < 0.0001$.

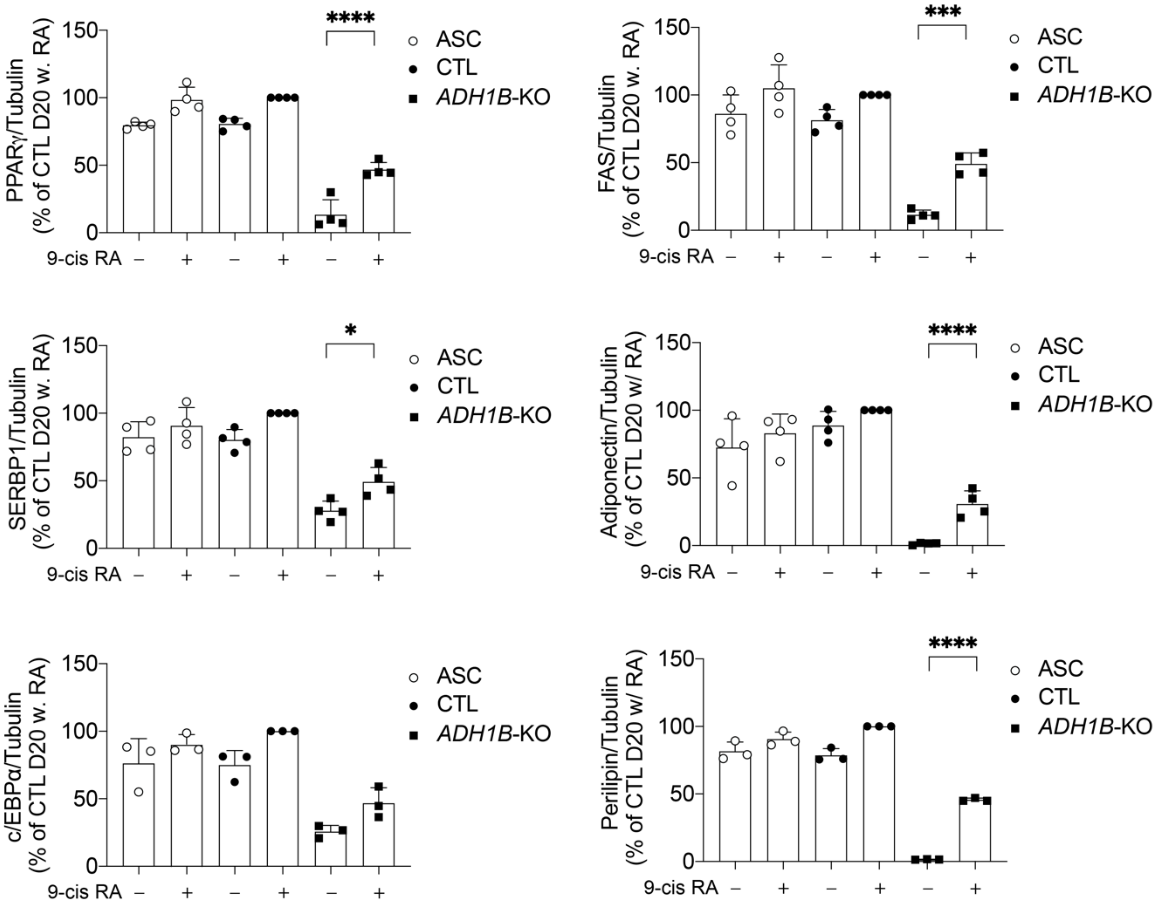

Fig. S4. Evaluation of adipocyte markers in control and edited ASC treated with 9-cis retinoic acid (RA). Data were obtained in ASC, ASC with a CRISPR-Cas9-mediated *ADH1B* knockout (KO), and ASC transduced with a Cas9/scramble gRNA plasmid corresponding to control (CTL) cells. The Western blots of adipocyte markers related to Figure 3E were quantified using FIJI software. *p*-values were determined by analysis of variance (ANOVA) with Bonferroni's *post hoc* multiple comparison test. $*p < 0.05$, $***p < 0.001$, and $****p < 0.0001$.

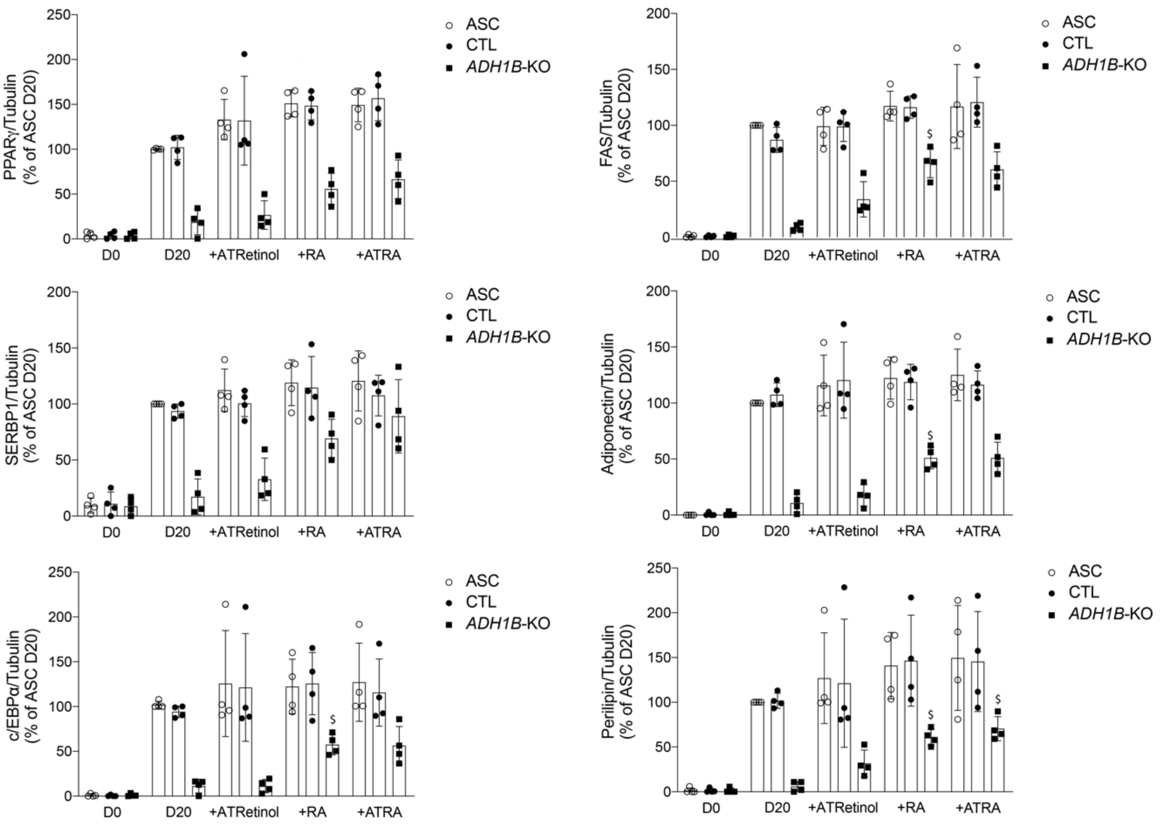

Fig. S5. Evaluation of adipocyte markers in control and edited ASC treated with 9-cis retinoic acid (RA), all-trans-retinol (ATRetinol) and all-trans-retinoic acid (ATRA). Data were obtained in ASC, ASC with a CRISPR-Cas9-mediated *ADH1B* knockout (KO), and ASC transduced with a Cas9/scramble gRNA plasmid corresponding to control (CTL) cells. The Western blots of adipocyte markers related to Figure 3I were quantified using FIJI software. *p*-values were determined by analysis of variance (ANOVA) with Brown-Forsythe and Welch's *post hoc* multiple comparison test. $ indicates that the difference is significant compared to ATRetinol.

Number of mismatches	Potential off-target sequences (mismatches are in red and bold characters)	Locus of the off-target (location, gene)
	**CCGCTCTTTACTCCTCAGTG (*ADH1B*)**	
3	**AT**GCTCTTTACTCC**A**CAGTG	intergenic, *RN7SKP141*
3	CCGCTCTTT**T**CTCCTCA**C**T**T**	intergenic, *DEPDC1*
3	CC**T**CTC**A**TT**G**CTCCTCAGTG	intron, *RP11-1080G15.2*
3	CC**C**CTC**A**TTACTCC**A**CAGTG	exon, *SMG7*
3	**AG**GCTCTTTA**A**TCCTCAGTG	intergenic, *WDR7-U3*
3	**AG**GCTCT**G**TACTCCTCAGTG	intergenic, *RBM47-NSUN7*
3	CC**C**CTCTTT**C**CTCCTCA**C**TG	intron, *SERPINE2*
3	**AAA**CTCTTTACTCCTCAGTG	intergenic, *GLUD2*
3	CC**A**CTCT**C**TACT**T**CTCAGTG	intron, *RPGRIP1L*
3	CC**C**CTCTT**CC**CTCCTCAGTG	intergenic, *VAC14*
3	**A**CG**T**TCTTTACTCCTCAGT**C**	intron, *AUTS2*
3	**T**C**CT**TCTTTACTCCTCAGTG	intron, *NTRK3*
3	CC**T**C**AG**TTTACTCCTCAGTG	intergenic, *PARD3B-NRP2*
0	CCGCTCTTTACTCCTCAGTG	exon, *ADH1B*

Table S1. List of predicted off-target sequences of the CRISPR/Cas9 editing strategy, with mismatch position and genomic location. The CRISPOR web tool (http://crispor.tefor.net/) is well recognized to predict the risk of off-target sequences by providing a cutting frequency determination (CFD) specificity score ranging from 1 to 100. The higher the number, the lower the risk of off-target effects. It is based on the accurate CFD off-target model from Doench JG et al. (Nat Biotechnol 2016 Feb;34(2):184-196), which recommends guides with a CFD specificity score > 50. The gRNA used herein to target *ADH1B* exon 4 has a CFD score of 86. All gRNA did not match perfectly any other genomic region. The table below provides a list of potential off-target sequences with up to three mismatches with the different gRNA used. Notably, off-targets are considered if they are flanked by an NGG motif, which corresponds to the PAM sequence allowing the Cas9 to cut DNA.

Dataset S1 (ppt file). Uncropped unedited blots.

Qatar Genome Project Consortium Authors

The following authors were part of the Qatar Genome Project Consortium:

Said I. Ismail[4], Wadha Al-Muftah[4], Radja Badji[4], Hamdi Mbarek[4], Dima Darwish[4], Tasnim Fadl[4], Heba Yasin[4], Maryem Ennaifar[4], Rania Abdellatif[4], Fatima Alkuwari[4], Muhammad Alvi[4], Yasser Al-Sarraj[4], Chadi Saad[4], Asmaa Althani[4,5], Eleni Fethnou[5], Fatima Qafoud[5], Eiman Alkhayat[5], Nahla Afifi[5], Sara Tomei[6], Wei Liu[6] & Stephan Lorenz[6], Najeeb Syed[7], Hakeem Almabrazi[7], Fazulur Rehaman Vempalli[7] & Ramzi Temanni[7], Tariq Abu Saqri[8], Mohammedhusen Khatib[8], Mehshad Hamza[8], Tariq Abu Zaid[8], Ahmed El Khouly[8], Tushar Pathare[8], Shafeeq Poolat[8], Rashid Al-Ali[8], Omar Albagha[3,17,20], Abdelaziz Belkadi[18], Souhaila Al-Khodor[9,20], Mashael Alshafai[10,20], Ramin Badii[11,20], Lotfi Chouchane[12,20], Xavier Estivill[13,20], Khalid Fakhro[1,2,3,14,20], Hamdi Mbarek[4,20], Younes Mokrab[1,2,3,15,20], Jithesh V. Puthen[3,20], Karsten Suhre[5,18,19,20], Zohreh Tatari[16,20]

[1] Department of Human Genetics, Sidra Medicine, Doha, Qatar
[2] Weill Cornell Medicine-Qatar, Doha, Qatar
[3] College of Health and Life Sciences, Hamad Bin Khalifa University, Doha, Qatar
[4] Qatar Genome Program, Qatar Precision Health Institute, Qatar Foundation, Doha, Qatar.
[5] Qatar Biobank for Medical Research, Qatar Precision Health Institute, Qatar Foundation, Doha, Qatar.
[6] Integrated Genomics Services, Sidra Medicine, Doha, Qatar.
[7] Applied Bioinformatics Core, Sidra Medicine, Doha, Qatar.
[8] Biomedical Informatics, Sidra Medicine, Doha, Qatar.
[9] Microbiome and Biomarkers Discovery lab, Sidra Medicine, Doha, Qatar.
[10] College of Health Sciences, Qatar University, Doha, Qatar.
[11] Molecular Genetics Lab, Hamad Medical Corporation, Doha, Qatar.
[12] Department of Genetic Medicine, Microbiology and Immunology, Weill Cornell Medicine-Qatar, Doha, Qatar.
[13] Research Branch, Sidra Medicine, Doha, Qatar.
[14] Genomic Medicine Lab, Sidra Medicine, Doha, Qatar.
[15] Medical and Population Genomics Lab, Sidra Medicine, Doha, Qatar.
[16] Clinical Research Centre, Sidra Medicine, Doha, Qatar.
[17] Centre for Genomic and Experimental Medicine, Institute of Genetics and Molecular Medicine, University of Edinburgh, Edinburgh, UK
[18] Bioinformatics Core, Weill Cornell Medicine-Qatar, Education City, Doha, Qatar
[19] Department of Biophysics and Physiology, Weill Cornell Medicine, New York, NY, USA
[20] Consortium lead principal investigators

The Qatar Genome Project Consortium coordinated the sampling, sequencing, and bioinformatic analysis for the Qatar Genome cohort.
