# Supplementary material for: ADH1B, the adipocyte-enriched alcohol dehydrogenase, plays an essential, cell-autonomous role in human adipogenesis
Source: Proc Natl Acad Sci U S A. 2024 Jun 5;121(24):e2319301121. doi: 10.1073/pnas.2319301121 (PMC11181076; doi:10.1073/pnas.2319301121)

Figure 1B

anti-ADH1B

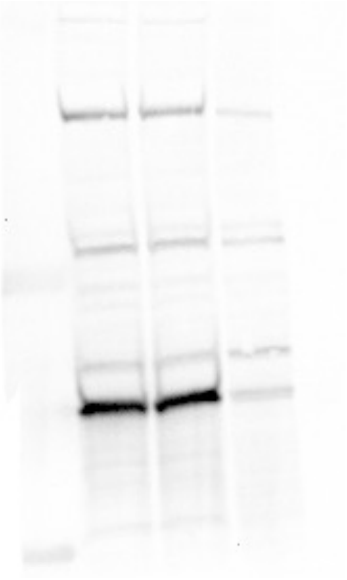

ASC at D0

anti-Tubulin

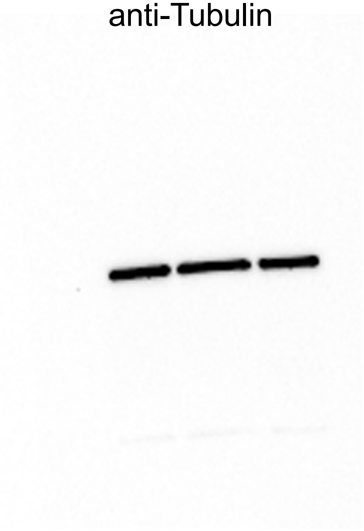

ASC at D0

Figure 1B

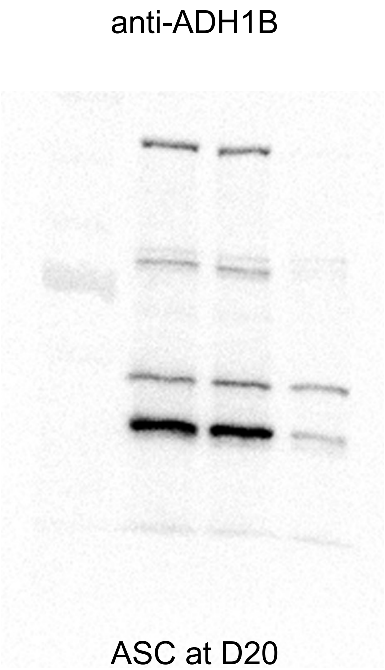

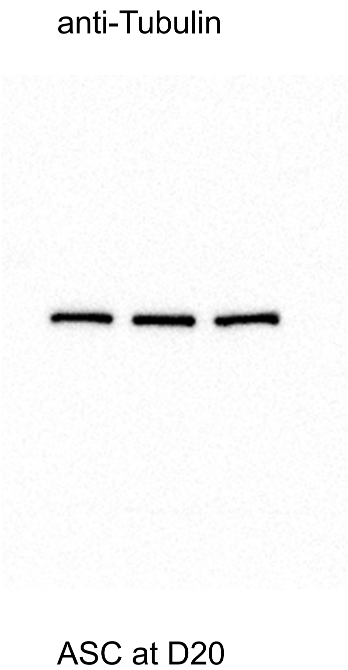

Figure 1F

anti-PPARγ

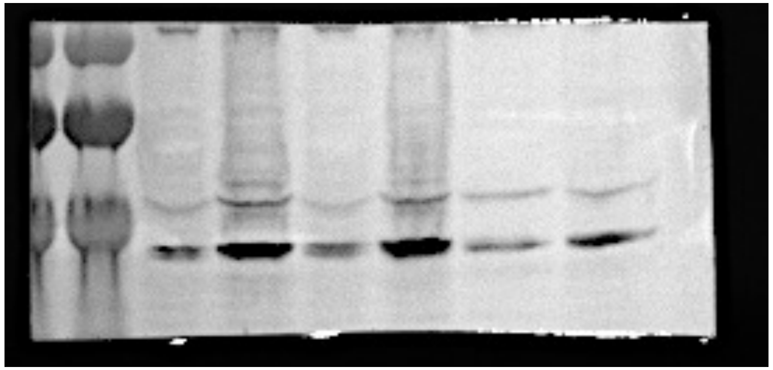

anti-SREBP1

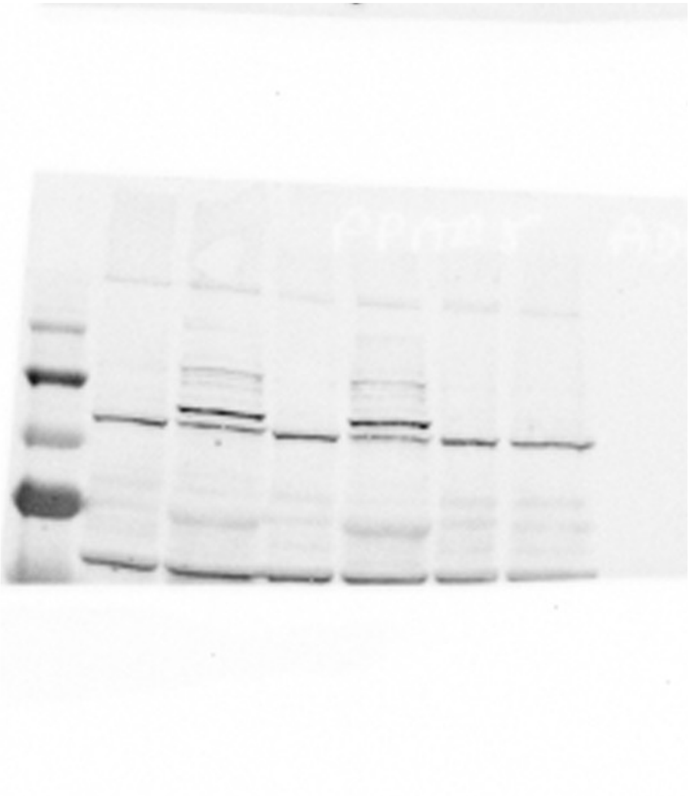

Figure 1F

anti-c/EBPα

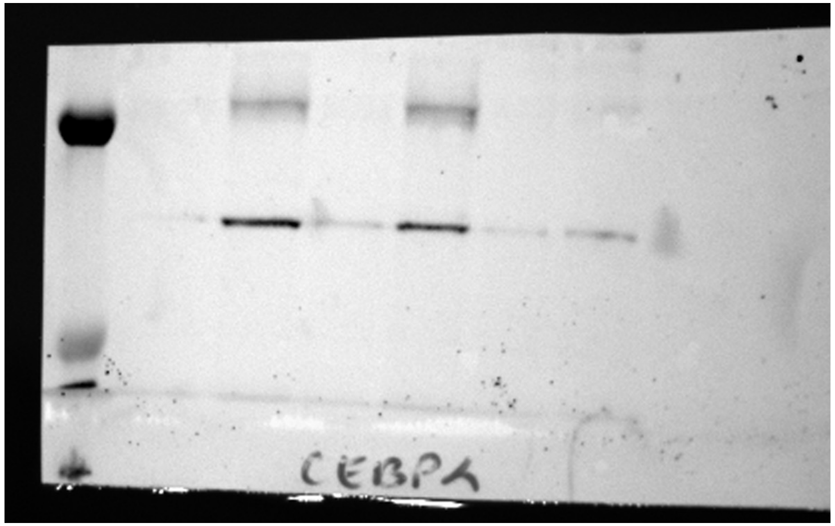

anti-FAS

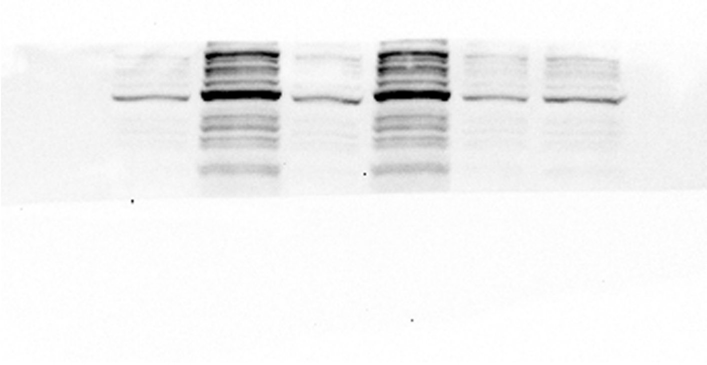

Figure 1F

anti-Adiponectin

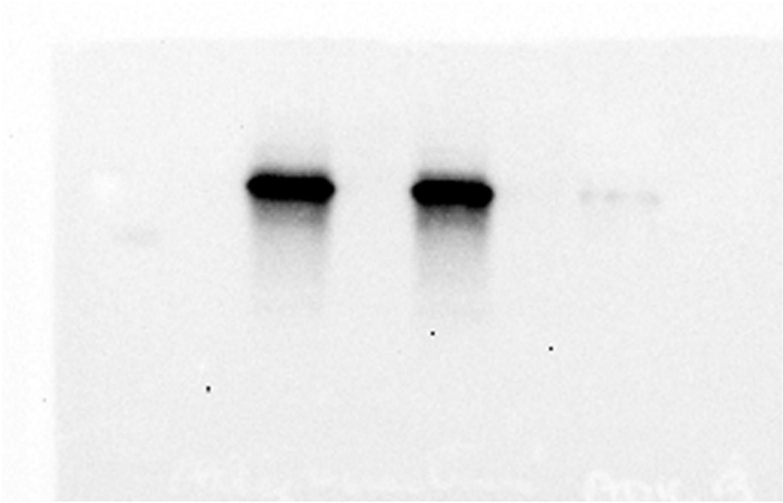

anti-Perilipin

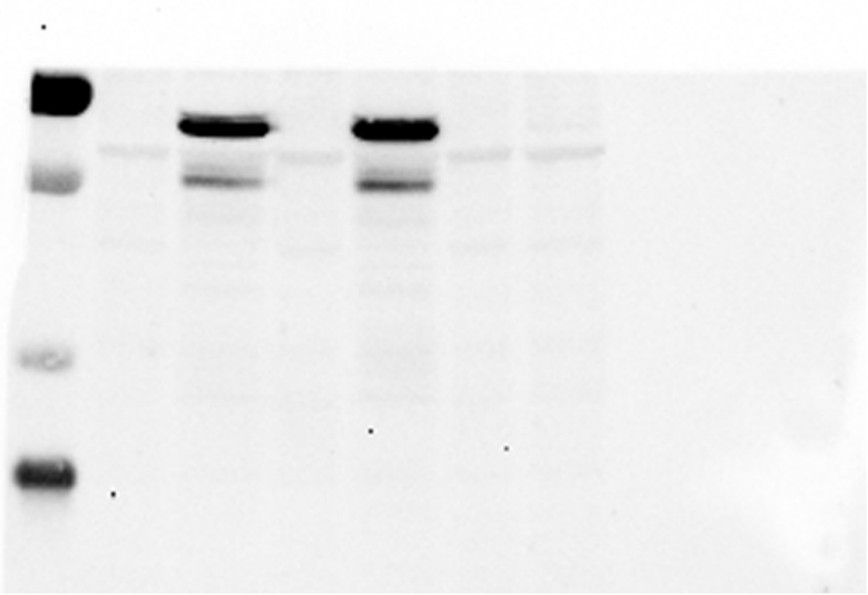

Figure 1F

anti-Leptin

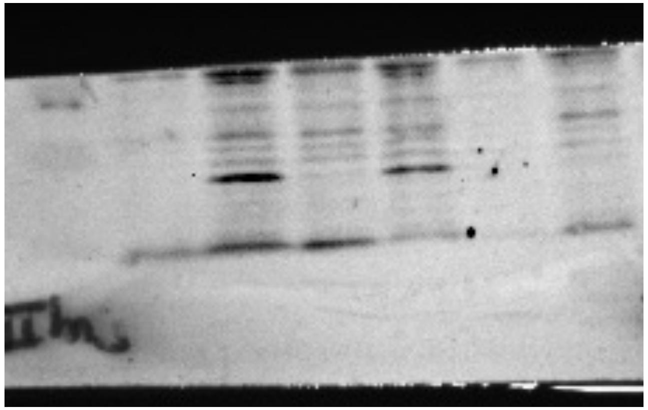

anti-Tubulin

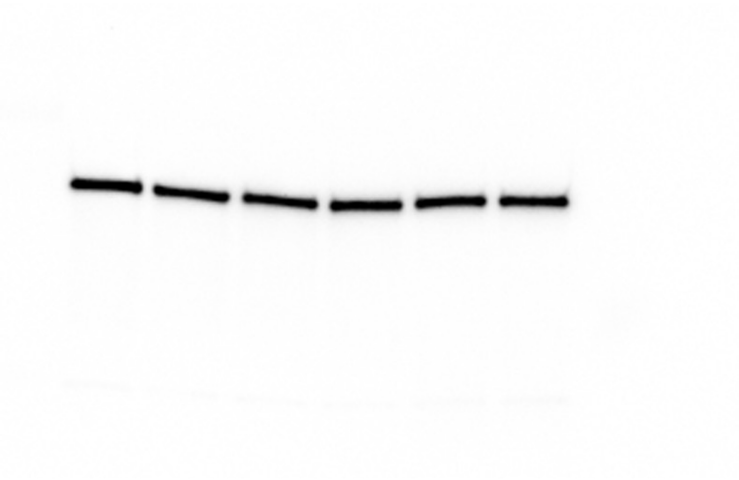

Figure 2E

anti-PPARγ

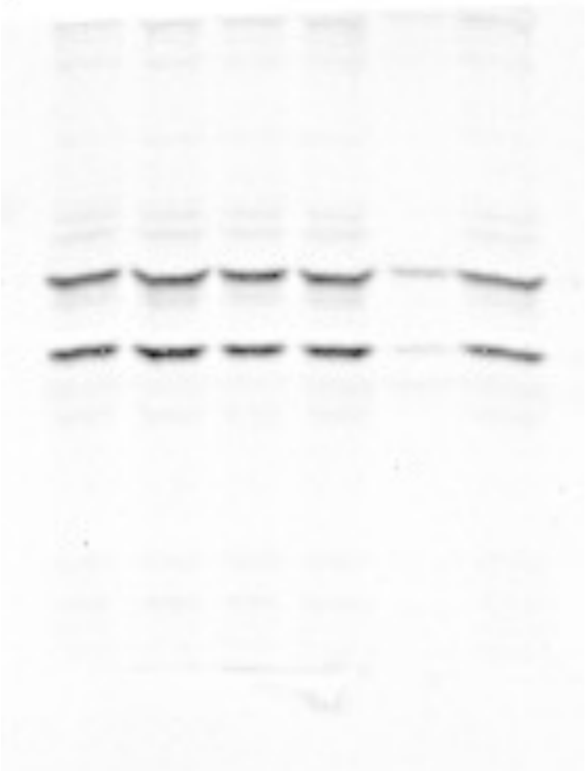

anti-SREBP1

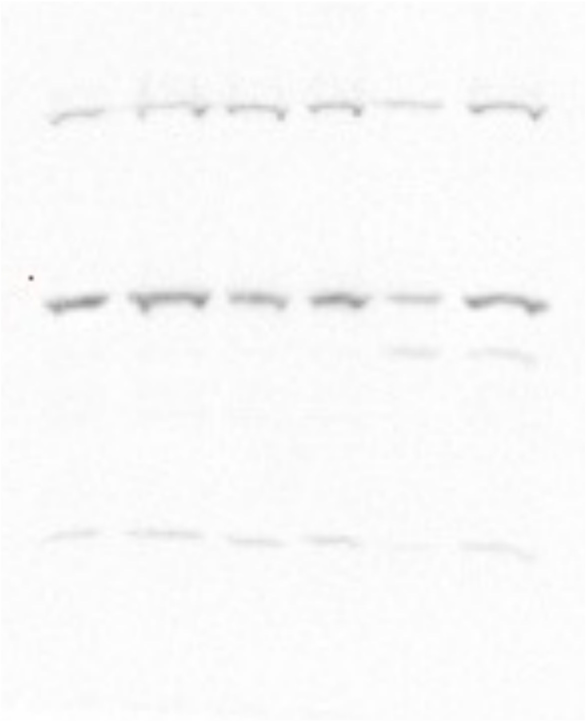

Figure 2E

anti-c/EBPα

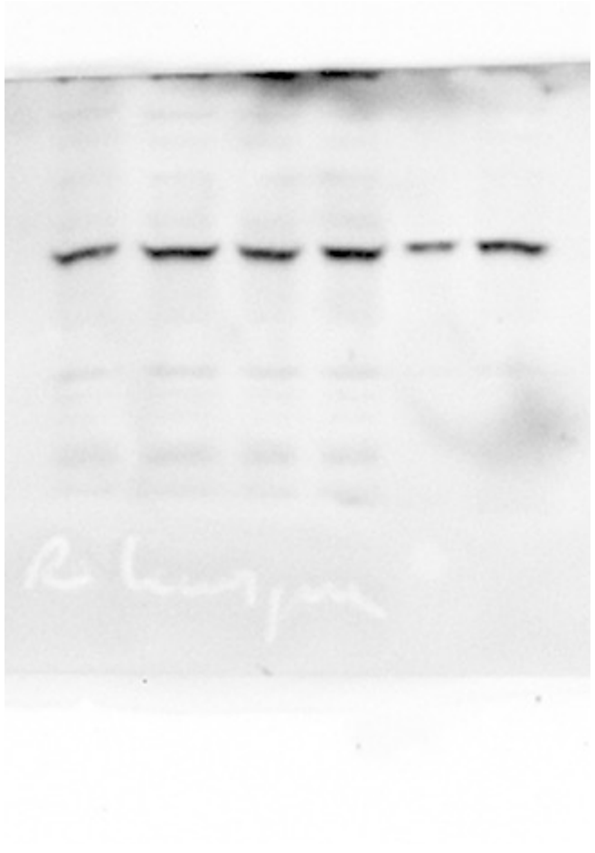

anti-FAS

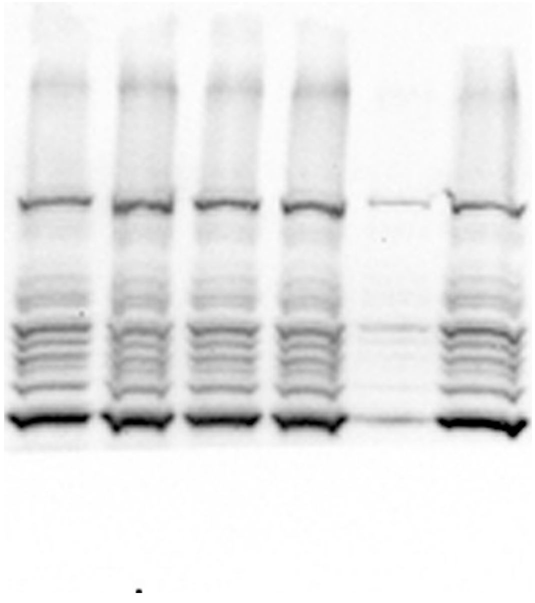

Figure 2E

anti-Adiponectin

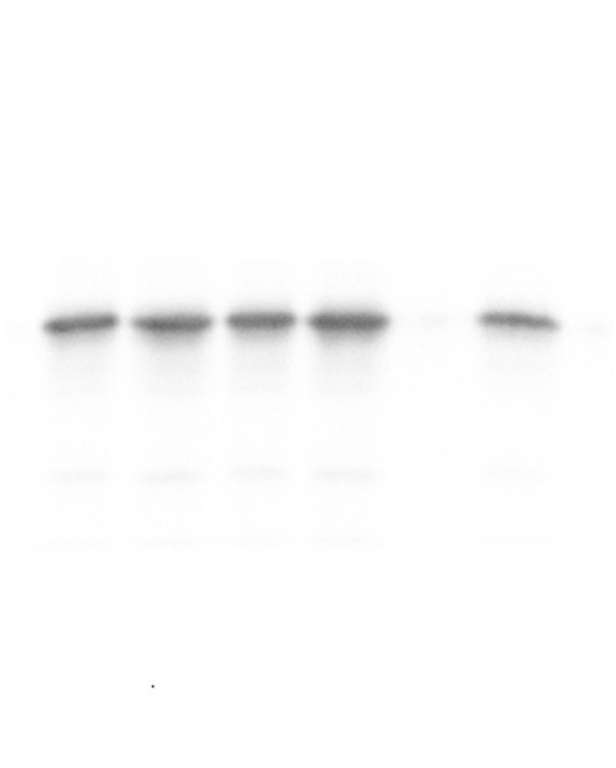

anti-Perilipin

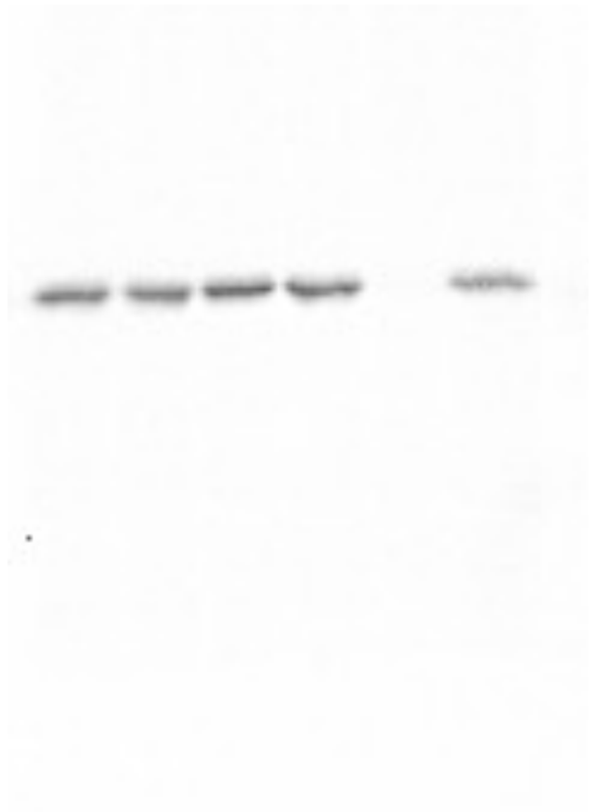

Figure 2E

anti-Tubulin

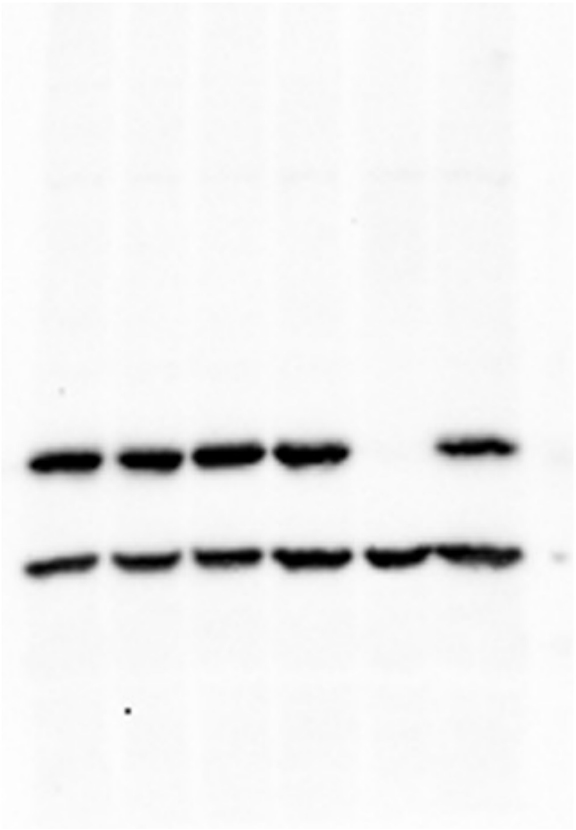

Figure 3D

anti-PPARγ

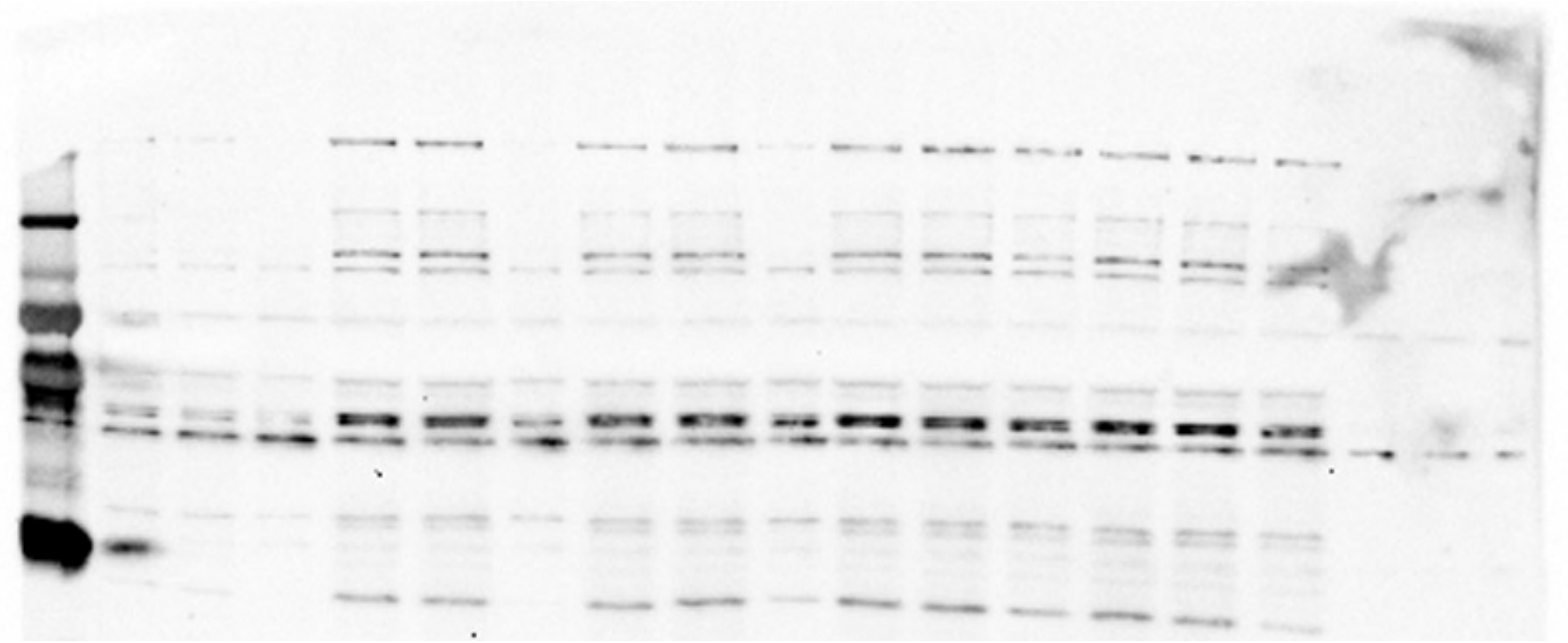

Figure 3D

anti-SREBP1

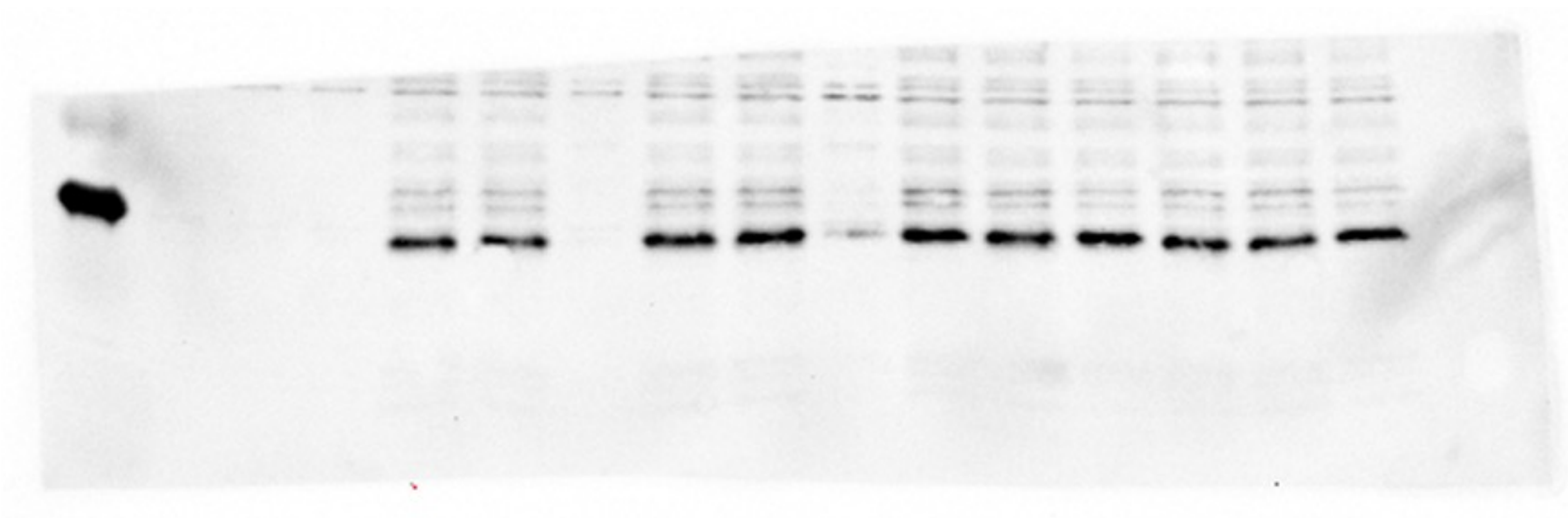

Figure 3D

anti-c/EBPα

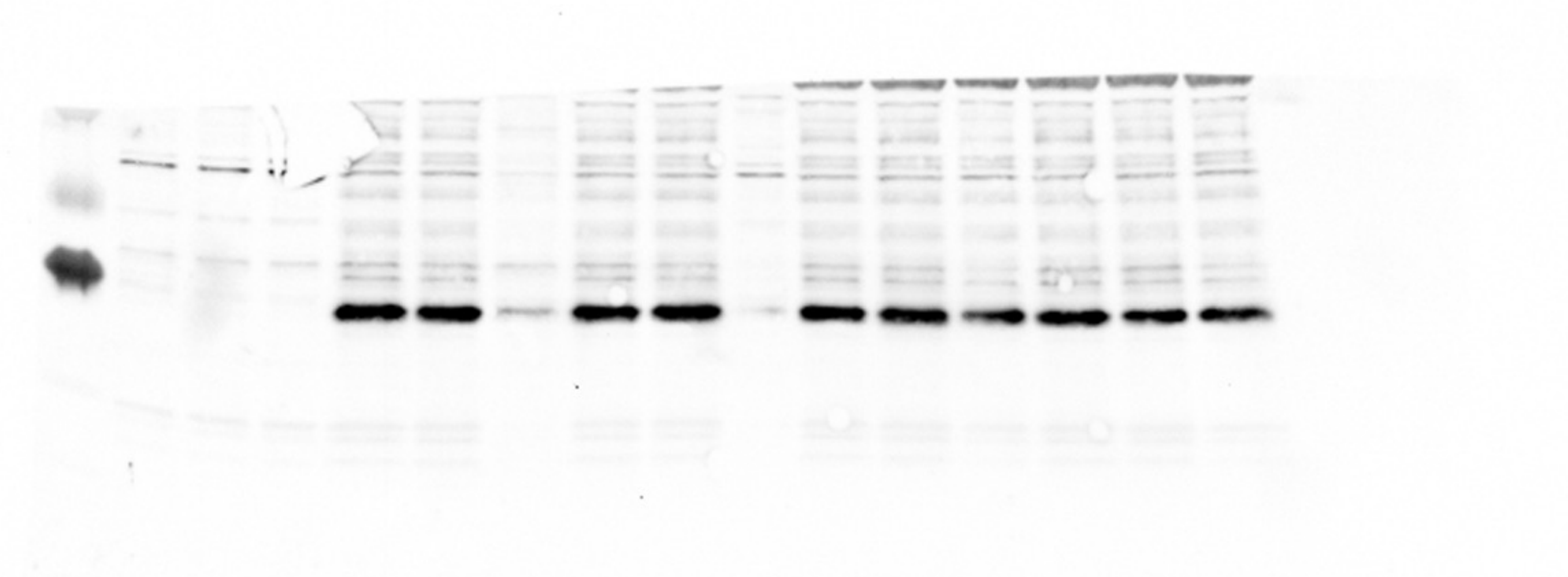

Figure 3D

anti-FAS

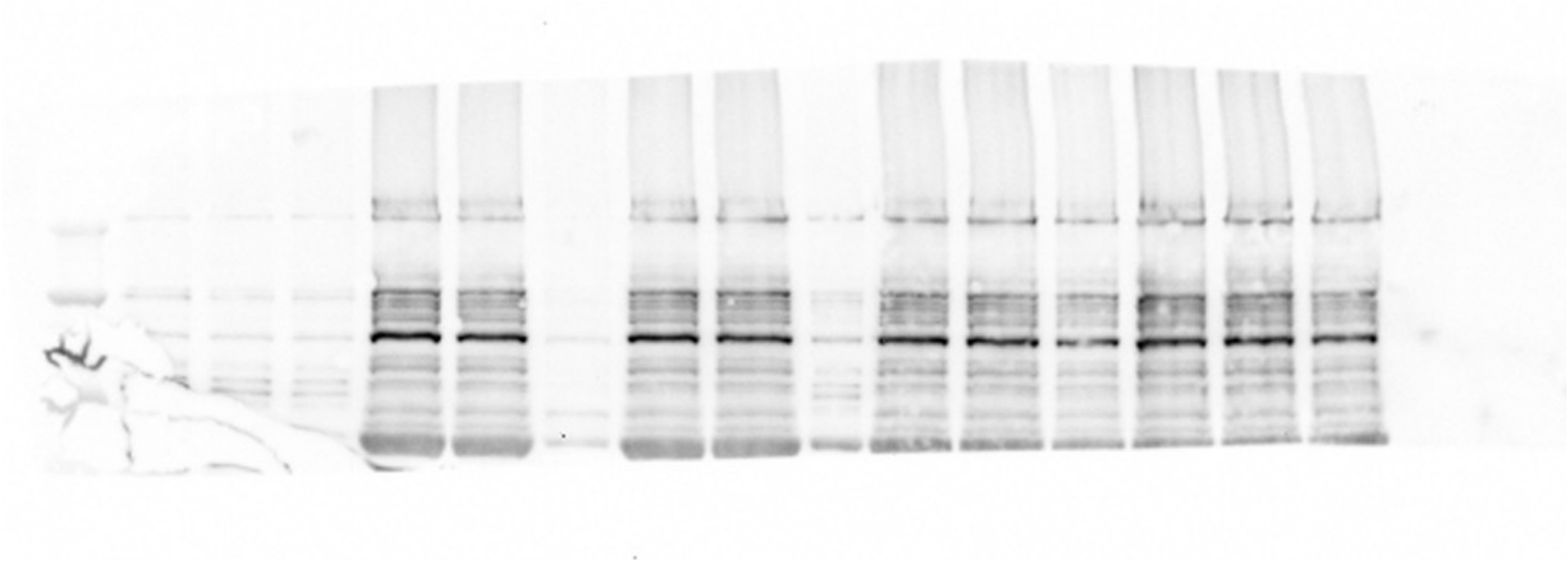

Figure 3D

anti-Adiponectin

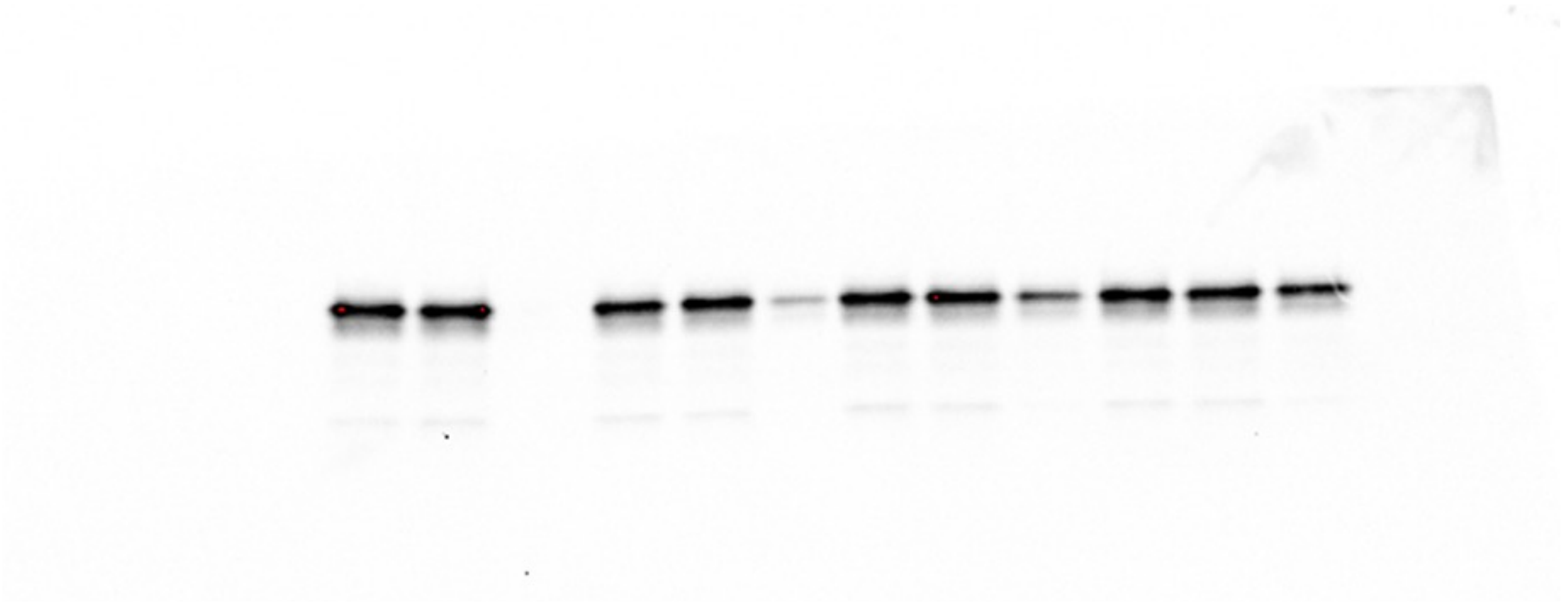

Figure 3D

anti-Perilipin

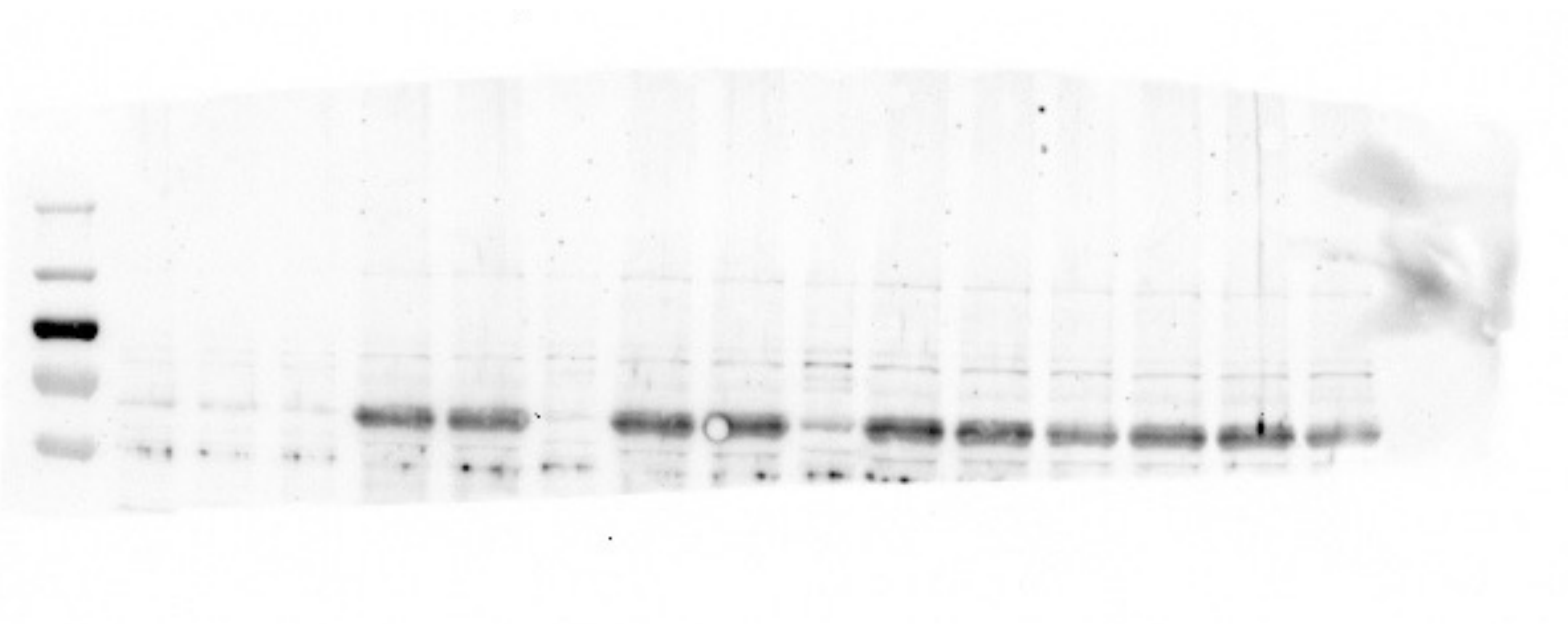

Figure 3D

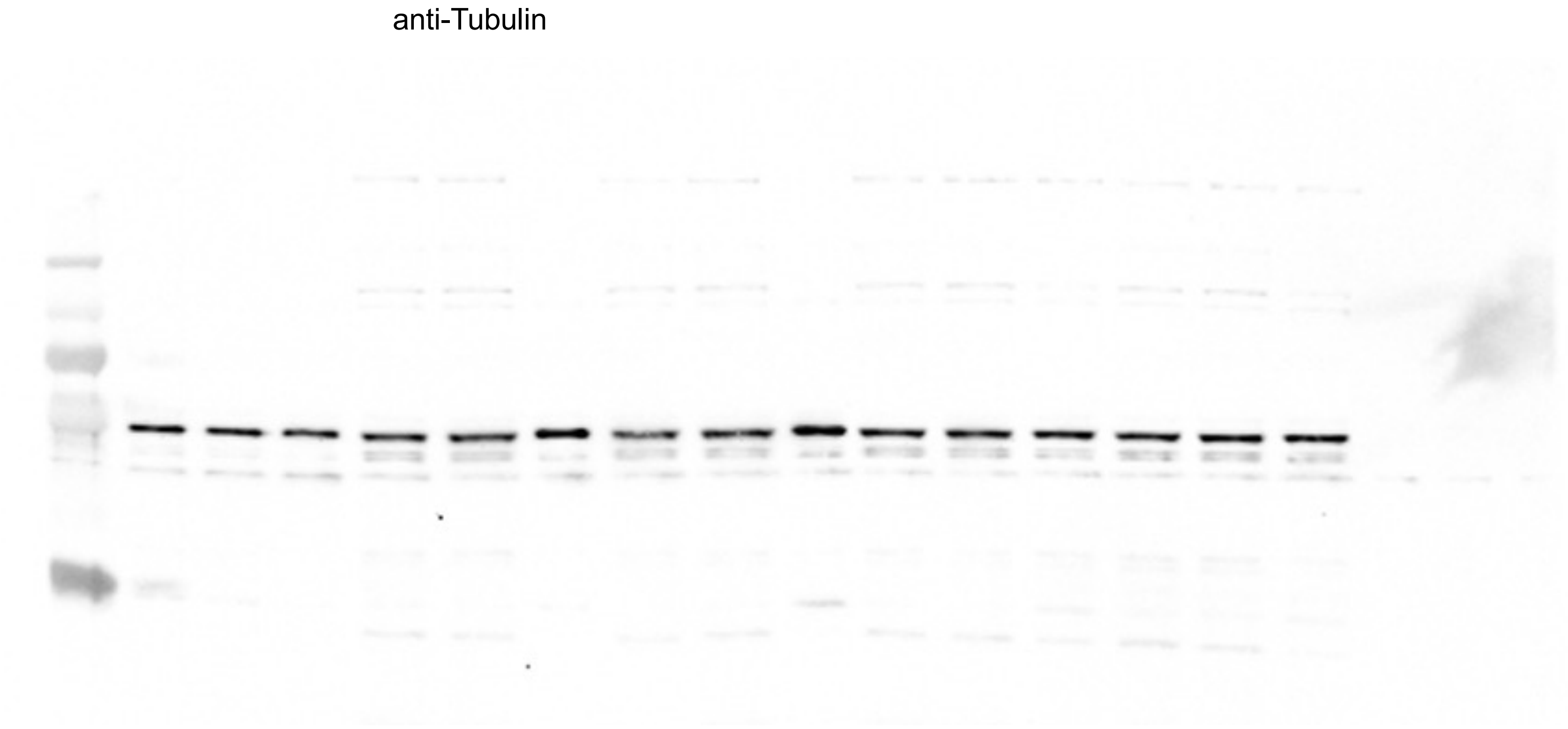

Figure 4F

anti-Flag (Native) anti-Flag (SDS) anti-Tubulin (SDS)

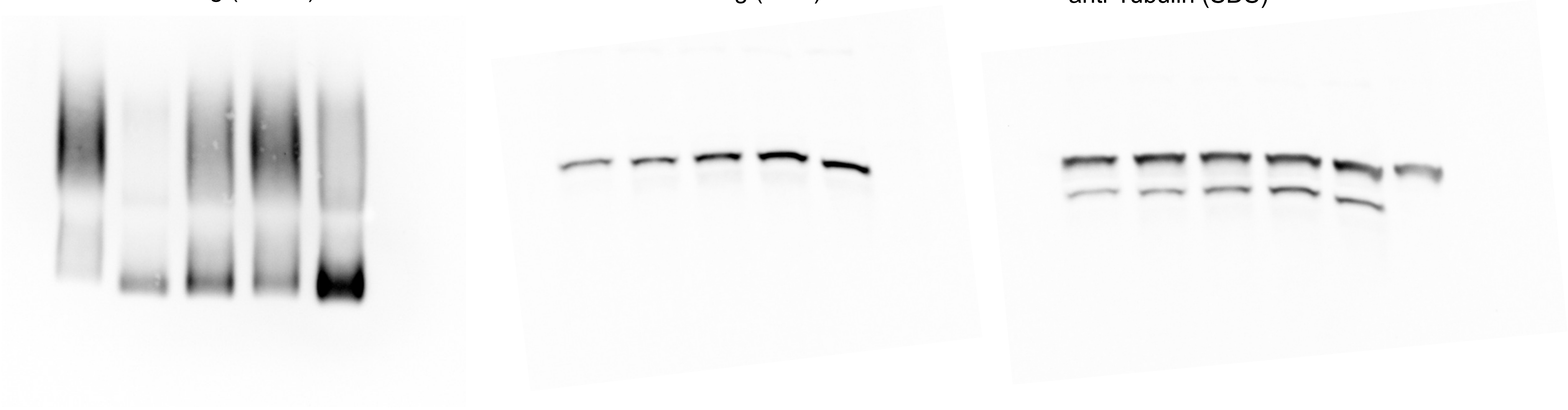

Figure 4H

anti-Flag

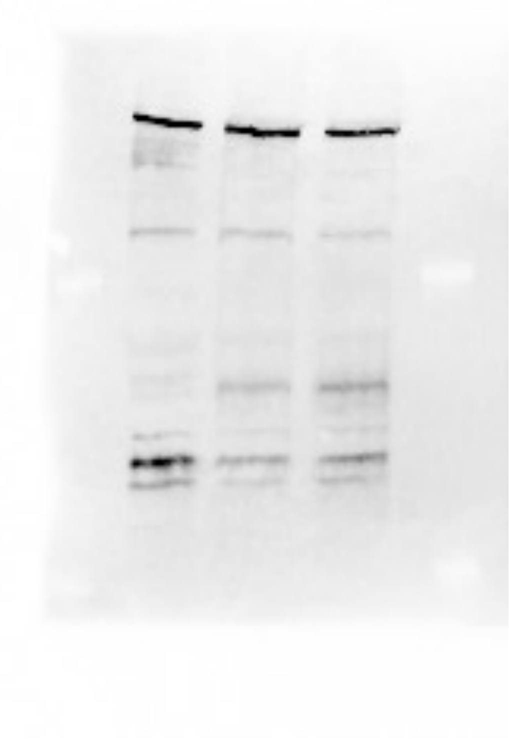

anti-Tubulin

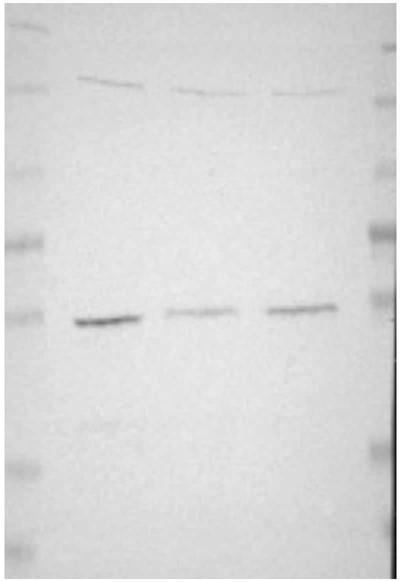

Supplement: Supplementary file 2 — Appendix 02 (PDF) [file pnas.2319301121.sapp02.pdf]
