# Supplementary material for: ADH1B, the adipocyte-enriched alcohol dehydrogenase, plays an essential, cell-autonomous role in human adipogenesis
Source: Proc Natl Acad Sci U S A. 2024 Jun 5;121(24):e2319301121. doi: 10.1073/pnas.2319301121 (PMC11181076; doi:10.1073/pnas.2319301121)
Supplement: Supplementary file 3 — Dataset S01 (DOCX) [file pnas.2319301121.sd01.docx]

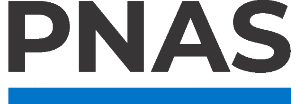


**Qatar Genome Project Consortium Authors**

The following authors were part of the Qatar Genome Project Consortium:

Said I. Ismail^4^, Wadha Al-Muftah^4^, Radja Badji^4^, Hamdi Mbarek^4^, Dima Darwish^4^, Tasnim Fadl^4^, Heba Yasin^4^, Maryem Ennaifar^4^, Rania Abdellatif^4^, Fatima Alkuwari^4^, Muhammad Alvi^4^, Yasser Al-Sarraj^4^, Chadi Saad^4^, Asmaa Althani^4,5^, Eleni Fethnou^5^, Fatima Qafoud^5^, Eiman Alkhayat^5^, Nahla Afifi^5^, Sara Tomei^6^, Wei Liu^6^& Stephan Lorenz^6^, Najeeb Syed^7^, Hakeem Almabrazi^7^, Fazulur Rehaman Vempalli^7^ & Ramzi Temanni^7^, Tariq Abu Saqri^8^, Mohammedhusen Khatib^8^, Mehshad Hamza^8^, Tariq Abu Zaid^8^, Ahmed El Khouly^8^, Tushar Pathare^8^, Shafeeq Poolat^8^, Rashid Al-Ali^8^, Omar Albagha^3,17,20^, Abdelaziz Belkadi^18^, Souhaila Al-Khodor^9,20^, Mashael Alshafai^10,20^, Ramin Badii^11,20^, Lotfi Chouchane^12,20^, Xavier Estivill^13,20^, Khalid Fakhro^1,2,3,14,20^, Hamdi Mbarek^4,20^, Younes Mokrab^1,2,3,15,20^, Jithesh V. Puthen^3,20^, Karsten Suhre^5,18,19,20^, Zohreh Tatari^16,20^

^1^ Department of Human Genetics, Sidra Medicine, Doha, Qatar

^2^ Weill Cornell Medicine-Qatar, Doha, Qatar

^3^ College of Health and Life Sciences, Hamad Bin Khalifa University, Doha, Qatar

^4^ Qatar Genome Program, Qatar Precision Health Institute, Qatar Foundation, Doha, Qatar.

^5^ Qatar Biobank for Medical Research, Qatar Precision Health Institute, Qatar Foundation, Doha, Qatar.

^6^ Integrated Genomics Services, Sidra Medicine, Doha, Qatar.

^7^ Applied Bioinformatics Core, Sidra Medicine, Doha, Qatar.

^8^ Biomedical Informatics, Sidra Medicine, Doha, Qatar.

^9^ Microbiome and Biomarkers Discovery lab, Sidra Medicine, Doha, Qatar.

^10^ College of Health Sciences, Qatar University, Doha, Qatar.

^11^ Molecular Genetics Lab, Hamad Medical Corporation, Doha, Qatar.

^12^ Department of Genetic Medicine, Microbiology and Immunology, Weill Cornell Medicine-Qatar, Doha, Qatar.

^13^ Research Branch, Sidra Medicine, Doha, Qatar.

^14^ Genomic Medicine Lab, Sidra Medicine, Doha, Qatar.

^15^ Medical and Population Genomics Lab, Sidra Medicine, Doha, Qatar.

^16^ Clinical Research Centre, Sidra Medicine, Doha, Qatar.

^17^ Centre for Genomic and Experimental Medicine, Institute of Genetics and Molecular Medicine, University of Edinburgh, Edinburgh, UK

^18^ Bioinformatics Core, Weill Cornell Medicine-Qatar, Education City, Doha, Qatar

^19^ Department of Biophysics and Physiology, Weill Cornell Medicine, New York, NY, USA

^20^ Consortium lead principal investigators

The Qatar Genome Project Consortium coordinated the sampling, sequencing, and bioinformatic analysis for the Qatar Genome cohort.
